# Mass reconstruction methods for PM_2.5_: a review

**DOI:** 10.1007/s11869-015-0338-3

**Published:** 2015-05-07

**Authors:** Judith C. Chow, Douglas H. Lowenthal, L.-W. Antony Chen, Xiaoliang Wang, John G. Watson

**Affiliations:** 1 Division of Atmospheric Sciences, Desert Research Institute, Reno, NV 89512 USA; 2 The State Key Laboratory of Loess and Quaternary Geology, Institute of Earth Environment, Chinese Academy of Sciences, Xi’an, Shaanxi 710075 China; 3 Graduate Faculty, University of Nevada, Reno, NV 89503 USA; 4 Department of Environmental and Occupational Health, University of Nevada, Las Vegas, NV 89154 USA

**Keywords:** PM_2.5_, Mass closure, Chemical speciation, Organic matter, Sampling artifact

## Abstract

Major components of suspended particulate matter (PM) are inorganic ions, organic matter (OM), elemental carbon (EC), geological minerals, salt, non-mineral elements, and water. Since oxygen (O) and hydrogen (H) are not directly measured in chemical speciation networks, more than ten weighting equations have been applied to account for their presence, thereby approximating gravimetric mass. Assumptions for these weights are not the same under all circumstances. OM is estimated from an organic carbon (OC) multiplier (*f*) that ranges from 1.4 to 1.8 in most studies, but *f* can be larger for highly polar compounds from biomass burning and secondary organic aerosols. The mineral content of fugitive dust is estimated from elemental markers, while the water-soluble content is accounted for as inorganic ions or salt. Part of the discrepancy between measured and reconstructed PM mass is due to the measurement process, including: (1) organic vapors adsorbed on quartz-fiber filters; (2) evaporation of volatile ammonium nitrate and OM between the weighed Teflon-membrane filter and the nylon-membrane and/or quartz-fiber filters on which ions and carbon are measured; and (3) liquid water retained on soluble constituents during filter weighing. The widely used IMPROVE equations were developed to characterize particle light extinction in U.S. national parks, and variants of this approach have been tested in a large variety of environments. Important factors for improving agreement between measured and reconstructed PM mass are the *f* multiplier for converting OC to OM and accounting for OC sampling artifacts.

**Electronic supplementary material:**

The online version of this article (doi:10.1007/s11869-015-0338-3) contains supplementary material, which is available to authorized users.

## Introduction

Particles with aerodynamic diameters <2.5 μm (PM_2.5_) and 10 μm (PM_10_) mass concentrations are regulated by the National Ambient Air Quality Standards (NAAQS; Bachmann 2007; Chow et al. 2007a) in the USA, with variations being adopted in other countries (Cao et al. 2013). For compliance monitoring, ambient particles are collected over 24-h durations onto filters that are weighed before and after sampling (Chow 1995; Watson and Chow 2011). Chemically speciated PM is needed to better understand pollution sources, atmospheric processing (e.g., transport and transformation), temporal and spatial variations and long-term trends, as well as adverse health and environmental consequences. PM_2.5_ mass and chemical components (i.e., ions, elements, and carbon) have been acquired in the National Park Service (NPS) Interagency Monitoring of Protected Visual Environments (IMPROVE) non-urban network, and the US Environmental Protection Agency (EPA) urban Chemical Speciation Network (CSN; Solomon et al. 2014; USEPA 2015) on an every-third- or sixth-day schedule since 1987/1988 and 1999/2000, respectively. Measurement protocols for the US PM_2.5_ networks are documented by Chow et al. (2010) and Solomon et al. (2014). Sampling and chemical analysis methods vary in these and other long-term networks and in special studies from the USA and elsewhere (e.g., Dabek-Zlotorzynska et al. 2011; Zhang et al. 2012).

Chow and Watson (2013) summarize different PM chemical analysis methods. The major PM components measured to explain gravimetric mass include: (1) anions (e.g., chloride (Cl^−^), nitrate (NO_3_^−^), and sulfate (SO_4_^=^)) and cations (e.g., water-soluble sodium (Na^+^), potassium (K^+^), and ammonium (NH_4_^+^)); (2) elements, including metals (up to 51 elements from sodium (Na) to uranium (U)); and (3) organic carbon (OC) and elemental carbon (EC) and their carbon fractions. To accommodate chemical speciation, at least two types of sampling substrates (i.e., Teflon-membrane and quartz-fiber filters) are needed (Chow 1995). IMPROVE and CSN use three parallel channels, in which mass by gravimetry and elements by X-ray fluorescence (XRF; Watson et al. 1999) are measured on Teflon-membrane filters; ions by ion chromatography (IC; Chow and Watson 1999) are measured on nylon-membrane filters preceded by a sodium carbonate (Na_2_CO_3_) denuder (Ashbaugh and Eldred 2004) to remove nitric acid (HNO_3_); and OC and EC by thermal/optical carbon analysis (Chow et al. 1993, 2007a, 2011) are measured on quartz-fiber filters. PM components include carbon (C), hydrogen (H), nitrogen (N), sulfur (S), oxygen (O), and a wide variety of other elements. Owing to practical analytical limitations (Chow and Watson 2013), most networks do not measure H and O associated with OC, geological minerals, and liquid water—with the exception of the IMPROVE network, where H was quantified from 1988 to 2010 (Nejedly et al. 1997). As a result, the sum of the measured species is often lower than the gravimetric mass. Watson (2004) specifies a percent mass explained of 100 ± 20 % for source apportionment models, and this is a reasonably good criteria for mass reconstruction.

PM mass reconstruction (also called mass closure or material balance) applies multipliers to several of the measured species to estimate unmeasured components. Mass reconstruction is used to: (1) identify and correct potential measurement errors as part of data validation efforts (Chow et al. 1994a; Malm et al. 2011; Watson et al. 2001); (2) understand temporal and spatial variations of chemical composition (Hand et al. 2014; Malm et al. 2011); and (3) estimate source contributions to PM and light extinction (Chow and Watson 2013; Watson 2002). Mass reconstruction attempts to achieve closure between gravimetric mass and the sum of major components with assumptions to account for unmeasured species, but without double counting. For example, when SO_4_^=^ is included, elemental S is omitted; inclusion of elemental chlorine (Cl) excludes water-soluble Cl^−^; and the same applies for elemental potassium (K) and water-soluble potassium (K^+^) (Chow et al. 1994a). Although this review focuses on PM_2.5_, a similar approach is applicable for PM_10_. As PM_2.5_ is part of PM_10_, mass reconstruction should be conducted for both PM_2.5_ and PM_coarse_ (i.e., PM_10–2.5_) when PM_10_ speciation is available (e.g., Chow et al. 2002a).

Various approaches have been taken for PM mass reconstruction (e.g., Frank 2006; Hand et al. 2011; Malm et al. 2011)—the widely used 11 equations are documented in “Commonly applied reconstructed mass equations.” Applications of these equations to past studies (summarized in the supplemental material) are enumerated in “Applications of mass reconstruction equations to special studies.” To provide a perspective on the fraction of mass explained, examples of mass reconstruction applications for the long-term US IMPROVE network are given in “Evaluation of mass reconstruction through analysis of large data sets.” Various regression techniques have been used to derive multipliers for major PM components and to examine the adequacy of using the IMPROVE equations for mass reconstruction. Major factors that bias mass reconstruction (e.g., the use of an OC multiplier to estimate organic matter (OM), carbon sampling and analysis artifact, ammonium and nitrate volatilization, and particle-bound water on Teflon-membrane filters) are discussed in “Major factors influencing mass reconstruction.” This review examined hundreds of prior studies and intends to: (1) track the evolution and approaches for mass reconstruction; (2) discuss the adequacy of each approach; and (3) address major PM sampling and analysis issues that influence mass reconstruction.

## Commonly applied reconstructed mass equations

Table 1 summarizes 11 PM mass reconstruction methods (i.e., Eqs. 1 to 11, sequence in chronological order of publication) that have been applied to data acquired since the late 1970s. Some variations from other studies are referenced. Reconstructed mass (RM) is expressed as the sum of its seven representative chemical components, including: (1) inorganic ions; (2) OM or OC; (3) EC, also referred to as “black carbon” (BC), “soot,” or light absorbing carbon (LAC); (4) geological minerals (or materials), often referred to as “dust,” “soil,” or “crustal material;” (5) salt (sea salt near oceans and inland seas, but also deriving from wintertime de-icing material and desert playas); (6) trace elements (other elements that are not accounted for as minerals, as from fly ash); and (7) “others,” or “remaining mass,” representing other unaccounted or unidentified components. As such, RM equations take the following form:

**Table 1:** Summary of the 11 mass reconstruction equations and their major chemical components

Equation No. (reference)/study area	Inorganic ions	Organic mass/organic carbon (OM/OC) ratio	Elemental carbon (EC)	Geological minerals^a^	Salt^b^	Trace elements^c^	Others
Equation 1 (Macias et al. 1981)/Page, AZ	(NH_4_)_2_SO_4_ + NH_4_NO_3_	1.5^d^	Yes	1.89Al + 2.14Si + 1.4Ca + 1.2K + 1.43Fe (assuming Al_2_O_3_, SiO_2_, CaO, K_2_O, and Fe_2_O_3_)	None	1.25Cu + 1.24Zn + 1.08Pb (assuming CuO, ZnO, and PbO)	None
Equation 2 (Solomon et al. 1989)/Los Angeles, CA	SO_4_^=^ + NO_3_^−^ + NH_4_^+^	1.4	Yes	1.89Al + 2.14Si + 1.4Ca + 1.43Fe (no oxides were specified)	None	Sum of all species measured by XRF (excluding S, Al, Si, Ca, and Fe) plus Na^+^ and Mg^++^ measured by AAS	None
Equation 3 (Chow et al. 1994b)/Los Angeles, CA	SO_4_^=^ + NO_3_^−^ + NH_4_^+^	1.4	Yes	As in Eq. 2 (assuming Al_2_O_3_, SiO_2_, CaO, and Fe_2_O_3_)	None	Sum of 40 elements (Na to U) by XRF excluding S, Al, Si, Ca, and Fe	None
Equation 4 (Malm et al. 1994)/IMPROVE network	4.125S as (NH_4_)_2_SO_4_	1.4	Yes	2.2Al + 2.49Si + 1.63Ca + 1.94Ti + 2.42Fe (assuming Al_2_O_3_, SiO_2_, CaO, Fe_2_O_3_, and FeO (in equal amounts), TiO_2_, and K_2_O (assuming that soil K is 0.6Fe), with all oxide multipliers by 1.16 to account for other missing compounds)	None	None	None
	NO_3_^−^ was excluded due to the concern that NO_3_^−^ can volatilize from the Teflon-membrane filters but not from the Nylon filter						
Equation 5 (Chow et al. 1996)/San Joaquin Valley, CA	SO_4_^=^ + NO_3_^−^ + NH_4_^+^	1.4	Yes	As in Eq. 2	Na^+^ + Cl^−^	As in Eq. 2: also excluding Na^+^, K^+^, and Cl^−^	None
Equation 6 (Andrews et al. 2000)/Great Smoky Mountains National Park, TN	SO_4_^=^ + NO_3_^−^ + NH_4_^+^	1.4	Yes	As in Eq. 2 plus 1.67Ti (assuming Al_2_O_3_, SiO_2_, CaO, K_2_O, TiO_2_, and Fe_2_O_3_)	None	Sum of remaining species (excluding S, Al, Si, Fe, Ti, Ca, and K; see Table S-1 of Andrews et al. 2000)	None
	(MOUDI sampler NH_4_^+^ was estimated by HEADS SO_4_^=^/NH_4_^+^ ratio)						
Equation 7 (Malm et al. 2000); original IMPROVE Eq.)/ IMPROVE network	4.125S (as (NH_4_)_2_SO_4_) + 1.29NO_3_^−^ (as NH_4_NO_3_)	1.4	Yes	As in Eq. 4	None	None	None
Equation 8 (Maenhaut et al. 2002)/Melpitz, Germany	SO_4_^=^ + NO_3_^−^ + NH_4_^+^	1.4	Yes	As in Eq. 4	Cl + 1.4486Na	Sum of all non-sea salt and non-crustal elements, excluding S and K.	Non-crustal K (K − 0.6Fe)
Equation 9 (DeBell et al. 2006)/IMPROVE network	4.125S (as (NH_4_)_2_SO_4_) + 1.29NO_3_^−^ (as NH_4_NO_3_)	1.8	Yes	As in Eq. 4	None	None	None
Equation 10 (Hand et al. 2011; revised IMPROVE Eq.)/IMPROVE network	1.375 SO_4_^=^ (as (NH_4_)_2_SO_4_)^e^ + 1.29NO_3_^−^ (as NH_4_NO_3_)	1.8	Yes	As in Eq. 4	1.8Cl^−^	None	None
Equation 11 (Simon et al. 2011)/IMPROVE network	(NH_4_)_2_SO_4_ + NH_4_NO_3_	1.8	Yes	3.48Si + 1.63Ca + 2.42Fe + 1.94Ti	1.8Cl^−^	None	Non-crustal K = 1.2 × (K − 0.6Fe)

*(NH*_*4*_ *)*_*2*_ *SO*_*4*_ ammonium sulfate, *NH*_*4*_ *NO*_*3*_ ammonium nitrate, *S* sulfur, *SO*_*4*_^*=*^ sulfate, *NH*_*4*_^*+*^ ammonium, *NO*_*3*_^*−*^ nitrate, *MOUDI*, Multi-Orifice Uniform Deposit Impactor, *HEADS* Harvard-EPA Annular Denuder System

^a^Geological minerals include: aluminum (Al), aluminum oxide (Al_2_O_3_), silicon (Si); silicon oxide (SiO_2_), potassium (K); potassium oxide (K_2_O); calcium (Ca); calcium oxide (CaO), titanium (Ti), titanium oxide (TiO_2_), iron (Fe), ferric oxide (FeO), and ferrous oxide (Fe_2_O_3_)

^b^Salt includes: sea salt, chloride (Cl^−^), potassium ion (K^+^), and sodium ion (Na^+^)

^c^Trace elements include: barium (Ba), chromium (Cr), copper (Cu), lead (Pb), vanadium (V), zinc (Zn), copper oxide (CuO), lead oxide (PbO), and zinc oxide (ZnO); measurement methods are X-ray fluorescence (XRF) and atomic absorption spectroscopy (AAS)

^d^Based on assumed organic compound composition proportional to CH_2_O_0.25_

^e^Hand et al. (2011) estimated (NH_4_)_2_SO_4_ from the SO_4_^=^ concentration as 1.375 × SO_4_^=^ to account for unmeasured NH_4_^+^

A $$ \mathrm{R}\mathrm{M} = \mathrm{Inorganic}\ \mathrm{ions} + \mathrm{O}\mathrm{M} + \mathrm{E}\mathrm{C} + \mathrm{Geological}\ \mathrm{minerals} + \mathrm{Salts} + \mathrm{Trace}\ \mathrm{elements} + \mathrm{O}\mathrm{thers} $$

Each of these components can derive from a variety of sources, though they are often dominated by a few sources. Minerals, for example, do not include OM that might be associated with engine exhaust or bioaerosols deposited onto roadways or agricultural soils. These would be included in the OM fraction. Similarly, some fugitive dust sources include salts, but these would be accounted for in the salt fraction; sulfates and nitrates that react with salt (Hoffman et al. 2004) would be accounted for in the inorganic ion fraction. The background and assumptions related to these RM components are described in the following subsections.

### Inorganic ions

In addition to commonly measured anions and cations by IC, automated colorimetric (AC), atomic absorption spectroscopy (AAS), and inductively coupled plasma-atomic emissions spectroscopy (ICP-AES) have also been applied for ionic speciation (Chow and Watson 2013). Depending on the measurements available, the following methods are used to determine their mass contributions:

- In the absence of NH_4_^+^ measurement, SO_4_^=^ and NO_3_^−^ are assumed to be neutralized to ammonium sulfate ((NH_4_)_2_SO_4_) and ammonium nitrate (NH_4_NO_3_), with the NH_4_^+^ fraction accounted for by stoichiometric multipliers: 1.375SO_4_^=^ and 1.29NO_3_^−^, respectively (i.e., Eqs. 1, 10, and 11 in Table 1). An ion balance based on molar equivalence between the measured anions and cations should be applied to verify the extent of neutralization (Chow et al. 1994b).
- SO_4_^=^, NO_3_^−^, and NH_4_^+^ are summed without weighting factors (i.e., Eqs. 2, 3, 5, 6, and 8). This does not account for H when SO_4_^=^ is incompletely neutralized by NH_4_^+^ as in sulfuric acid (H_2_SO_4_), ammonium bisulfate (NH_4_HSO_4_), or letovicite ((NH_4_)_3_H(SO_4_)_2_).
- When only S is measured, it is assumed to be neutralized (NH_4_)_2_SO_4_ (i.e., 4.125S in Eqs. 7 and 9) and summed with either NO_3_^−^ (Landis et al. 2001) or NH_4_NO_3_ (1.29NO_3_^−^ in Eqs. 7 and 9). If NO_3_^−^ is not measured, NH_4_NO_3_ is assumed to be negligible (Malm et al. 1994, Eq. 4). This assumption is valid only when the NO_3_^−^ concentration is low, as it is for some non-urban, eastern US IMPROVE sites but not for others (Pitchford et al. 2009). Abundant NO_3_^−^ has been found in several urban areas, especially during fall and winter (Green et al. 2015).

Assuming 1.29NO_3_^−^ for NH_4_NO_3_ may not be valid when HNO_3_ reacts with suspended dust to form calcium nitrate (Ca(NO_3_)_2_) or when it reacts with sodium chloride (NaCl) from a marine intrusion or suspension from an alkaline playa to form sodium nitrate (NaNO_3_) (Hoffman et al. 2004). Lee et al. (2008) noted the presence of PM_2.5_ Ca(NO_3_)_2_ at several IMPROVE sites owing to a coarse particle NO_3_^−^ tail that extended below 2.5 μm. Harrison et al. (2003) applied Eq. 7 for PM_2.5_ ions and added NaNO_3_ for PM_10–2.5_. Several studies used front filter NO_3_^−^ (i.e., non-volatilized NO_3_^−^ from Teflon-membrane or quartz-fiber filters), as volatilized NO_3_^−^ is not part of the gravimetric mass (Chow et al. 2002a). Ma et al. (2001) estimated NH_4_NO_3_ as 2.857 N, with N measured by an elemental analyzer, which is commonly applied to fuel assays. The presence of ammonium chloride (NH_4_Cl) in PM_2.5_ was noted by Kelly et al. (2013) for Utah’s Salt Lake valley; by Pant et al. (2015) in New Delhi, India, where there is abundant trash burning; and by Levin et al. (2010) for biomass burning samples.

Elemental S has been commonly measured by XRF or proton-induced X-ray emission (PIXE) analyses (Watson et al. 1999). Based on molecular weight, 3S can be used to estimate SO_4_^=^, assuming that all S is water-soluble SO_4_^=^. This is not the case when: (1) S is associated with insoluble organic compounds such as mercaptans; (2) S is not completely water-soluble, as is the case for minerals such as gypsum (CaSO_4_·2H_2_O) and pyrite (FeS_2_); or (3) S consists of sulfur dioxide (SO_2_) adsorbed onto soot or other particles (Watson 2002).

For coastal environments, non-sea-salt sulfate (i.e., nssSO_4_^=^ = SO_4_^=^ − 0.252Na^+^, based on SO_4_^=^/Na^+^ molar ratio in sea water) can be estimated (Sciare et al. 2003). Summed nssSO_4_^=^ + NO_3_^−^ + NH_4_^+^ has been applied to estimate contributions from inorganic ions (Cheung et al. 2011; Maenhaut et al. 2008; Mkoma et al. 2009; Querol et al. 2001; Terzi et al. 2010). Zhang et al. (2013) also included K^+^ (a marker for biomass burning) as an additional inorganic ion.

Since NH_4_^+^ is not quantified in the IMPROVE network, (NH_4_)_2_SO_4_ is estimated by 4.125S (Eq. 7). Due to variations between SO_4_^=^ (by IC) and S (by XRF) ratios, Hand et al. (2011) used 1.375SO_4_^=^ (Eq. 10). Both the original (Eq. 7) and the revised (Eq. 10) IMPROVE equations have been the foundation for reconstructing light extinction in the USA under the Regional Haze Rule (now termed the Clean Air Visibility Rule; Pitchford et al. 2007; USEPA 2001; Watson 2002).

### Organic mass/organic carbon (OM/OC)

To account for the unmeasured H, O, N, and S in organic compounds, a conversion factor (or multiplier) is used to transform OC to OM, i.e.,

B $$ \mathrm{O}\mathrm{M} = f \times \mathrm{O}\mathrm{C} $$

The *f* multipliers of 1.4 and 1.8 in Table 1 are not site or time specific. Depending on the extent of OM oxidation and secondary organic aerosol (SOA) formation, values for *f* vary from 1.2 for fresh aerosol in urban areas (Chow et al. 2002a, b) to 2.6 for aged aerosol (Countess et al. 1980; Robinson et al. 2007, 2010; Roy et al. 2011; Turpin and Lim 2001). For example, benzo(a)pyrene (C_20_H_12_), an indicator of incomplete fuel combustion found in engine exhaust (Lowenthal et al. 1994) has an *f* = 1.05; whereas cellulose (C_6_H_10_O_5_)_*n*_, a major component of unburned biological material, has an *f* = 2.25 (Cerqueira et al. 2010; Puxbaum and Tenze-Kunit 2003; Sanchez-Ochoa et al. 2007).

The origins for *f* = 1.2–1.5 result from circular reasoning with limited measurements. Macias et al. (1981, Eq. 1) used 1.5 based on an assumed organic composition proportional to CH_2_O_0.25_. Solomon et al. (1989, Eq. 2) used 1.4, citing Gray et al. (1986), who used both 1.2 and 1.4 for studies in California’s South Coast Air Basin (SoCAB). The *f* = 1.2 originated from Countess et al. (1980), based on the analysis of ambient carboxylic acid (C_16_: (C + H + O)/C = 1.3), polynuclear aromatic ((C + H)/C = 1.08), and aliphatic compounds ((C + H)/C = 1.17) (van Vaeck and van Cauwenberghe 1978) in Denver, CO. Ma et al. (2001) used 1.4 but cited Countess et al. (1980). As noted by Andrews et al. (2000) and Watson (2002), the 1.4 derives from Grosjean and Friedlander (1975), based on two Los Angeles total suspended particle (TSP) samples. The ratios of C to the sum of C, H, N, and O was 0.66 for oxygenated organics and 0.86 for aliphatics; the inverses are 1.5 and 1.2, respectively. Gray et al. (1986) referred to White and Roberts (1977), who used *f* = 1.4 to construct a chemical light extinction budget based on Grosjean and Friedlander (1975). Harrison et al. (2003) used 1.4 for urban background sites in Birmingham, UK, and 1.3 for roadside sites in London, UK, citing Russell (2003).

Chow et al. (1994b; 1996, Eqs. 3 and 5, respectively) used 1.4, citing Solomon et al. (1989). Andrews et al. (2000, Eq. 6) also used 1.4, citing both White and Roberts (1977) and Grosjean and Friedlander (1975). Maenhaut et al. (2002, Eq. 8) used 1.4 for samples from Melpitz, Germany, citing Turpin et al. (2000). DeBell et al. (2006, Eq. 9) and Hand et al. (2011, Eq. 10) increased the *f* from 1.4 to 1.8 for the revised IMPROVE equation (Eq. 10) based on non-urban aerosols (e.g., El-Zanan et al. 2005) and regression analysis by Malm and Hand (2007). The average regression coefficient was 1.7 for OC across all IMPROVE sites for years 1988–2003. This is similar to the *f* = 1.8 used by Maenhaut et al. (2008) for samples from K-puszta, an EUSAAR station in Hungary, and by Mkoma et al. (2009) for a rural site in East Africa.

Several studies (e.g., Mkoma et al. 2009; Ni et al. 2013; Remoundaki et al. 2013; Terzi et al. 2010; Vecchi et al. 2008; Viana et al. 2007) used an *f* multiplier of 1.6, whereas *f* = 1.7 was reported by others (e.g., Guinot et al. 2007; Putaud et al. 2000; Rees et al. 2004). The value of the *f* multiplier under different situations remains the subject of current research. Biomass burning (especially during the smoldering phase) may require a higher *f* multiplier as it contains many oxygenated organic compounds (Chen et al. 2010; Chow et al. 2007b), such as levoglucosan (C_6_H_10_O_5_), a wood smoke marker (Simoneit et al. 1999) with the same chemical formula but a structure that differs from cellulose. For laboratory-generated vegetative burning, Levin et al. (2010) reported *f* = 1.55, consistent with a finding of *f* = ∼1.5 by Reid et al. (2005). Aiken et al. (2008) reported *f* = 1.55–1.7 for primary biomass combustion emissions in Mexico City, lower than 1.9–2.1 found by Polidori et al. (2008) in Pittsburgh, PA, during winter and 2.2–2.6 suggested by Turpin and Lim (2001).

### Elemental carbon

The RM equation in Table 1 contain EC without any multiplier. Since OC and EC are operationally defined, absolute OC and EC concentrations and the ratio of OC to EC vary by carbon analysis method (Watson et al. 2005).

### Geological minerals

Geological “minerals” might better represent geological “material,” as only assumed oxides of mineral elements (e.g., aluminum (Al), silicon (Si), calcium (Ca), K, titanium (Ti), and iron (Fe)) are included to calculate geological mass. These elements have been measured by XRF, PIXE (e.g., Maenhaut et al. 2008), and, in some cases, instrumental neutron activation analysis (INAA; Maenhaut et al. 2001; Siddique and Waheed 2014) or ICP-mass spectrometry (ICP-MS). Most researchers use one of the five soil formulae listed in Table 1. Macias et al. (1981, Eq. 1) expressed minerals as the sum of the oxides of Al, Si, Ca, K, and Fe assuming the common oxide forms of Al_2_O_3_, SiO_2_, CaO, K_2_O, and Fe_2_O_3_, respectively (Pettijohn 1975). Several studies eliminated the 1.2 K (Eq. 2), except for Andrews et al. (2000, Eq. 6), Kleindienst et al. (2010), and Ni et al. (2013), which also included 1.67Ti. A higher value (1.95Ca) was used by Terzi et al. (2010) and Remoundaki et al. (2013) to account for both CaO and CaCO_3_.

The IMPROVE “soil” formula (Malm et al. 1994, Eq. 4), applied in Eqs. 7–10, follows Macias et al. (1981, Eq. 1) with the following modifications: (1) iron oxides are equally divided between Fe_2_O_3_ and FeO; (2) K in soil is estimated as 0.6Fe, based on the composition of coarse particles (Cahill et al. 1986), because some PM_2.5_ K is emitted by biomass burning; and (3) titanium dioxide (TiO_2_) is included. All of the initial element coefficients are then multiplied by 1.16 to account for unmeasured O, therefore:

C $$ \mathrm{Geological}\ \mathrm{minerals} = 2.2\mathrm{A}\mathrm{l} + 2.49\mathrm{S}\mathrm{i} + 1.63\mathrm{C}\mathrm{a} + 1.94\mathrm{T}\mathrm{i} + 2.42\mathrm{F}\mathrm{e} $$

The IMPROVE “soil” formula (Eq. C) has been applied in several other studies (e.g., Chan et al. 1997; Pant et al. 2015). Rogula-Kozlowska et al. (2012) applied Eq. C but supplemented with 2.4K based on the stoichiometric concentration of K_2_O. Due to the uncertainties associated with Al by XRF (McDade 2008), Simon et al. (2011, Eq. 11) eliminated Al and used 3.48Si, based on the Al to Si ratio (0.46) in IMPROVE samples. Landis et al. (2001) also eliminated Al but used 3.79Si, citing uncertainties in quantifying Al by energy-dispersive XRF. Hueglin et al. (2005) estimated Si in Eq. 1 as 3.41Al (Mason 1966) and also included 1.66Mg.

Single crustal elements have also been used to estimate the geological mineral contribution to PM mass. Si is the most abundant element (10–20 %) in the earth’s crust besides O (Chow et al. 2003; Houck et al. 1989). Countess et al. (1980) used 3.5Si, and Ma et al. (2001) used 4.807Si (assuming 20.8 % Si in soil; Scheff and Valiozis 1990). Using Al as a soil marker (Duce et al. 1980), Ho et al. (2006) used 13.77Al, Hsu et al. (2008) used 12.5Al, and Zhang et al. (2013) used 14.29Al. Besides 4.3Ca (from gypsum), Harrison et al. (2003) used the sum of 9Fe for background and 3.5Fe to 5.5Fe for roadside sites, assuming 11–29 % of Fe in fugitive dust. Putaud et al. (2000) summed non-sea-salt (nss)K^+^, nssCa^++^, and gravimetric analyses of water insoluble species as residues (600 °C for 8 h) to estimate minerals. Since geological minerals are not a major component of PM_2.5_, variations in the assumptions regarding metal oxides or multipliers do not contribute to large variations in RM.

### Salt

Chow et al. (1996, Eq. 5) and Rogula-Kozlowska et al. (2012) used the sum of Na^+^ and Cl^−^ to track summertime transport of marine aerosol in California. Others (e.g., Maenhaut et al. 2002, Eq. 8, 2008; Mkoma et al. 2009; Viana et al. 2007) used Cl + 1.4486Na, based on the ratio of the sum of all elements (except Cl) to Na in sea water (Riley and Chester 1971). Ohta and Okita (1994) used 3.27Na^+^, and others (e.g., Chan et al. 1997; Chow et al. 2007a; Ho et al. 2006; Siddique and Waheed 2014; Yan et al. 2012) used 2.54Na^+^, whereas Harrison et al. (2003) and Joseph et al. (2012) used 1.65Cl^−^ to represent salt content.

PM_2.5_ Na is a conservative marker for salt (Lowenthal and Kumar 2006; White 2008), but it suffers self-absorption interferences by XRF (Dzubay and Nelson 1975; Formenti et al. 2010; Watson et al. 1999). Therefore, 1.8Cl^−^, based on the abundance of Cl^−^ in sea water (White 2008), is used in the revised IMPROVE equation (Eq. 10). This approach is reasonable when: (1) there is no depletion of Cl^−^ in salt aerosols from reaction with H_2_SO_4_ or HNO_3_; (2) hydrochloride acid (HCl) is retained on the nylon-membrane filter, i.e., the preceding Na_2_CO_3_ denuder to remove HNO_3_ (Channel 2 of the IMPROVE sampler) does not remove any HCl; and (3) HCl only originated from reactions of acids with salt particles. In any case, 1.8Cl^−^ is a lower limit to estimate salt, assuming that Cl^−^ is measured accurately by IC (Chow and Watson 1999). With advances in chromatographic techniques, the Cl^−^ signal in the chromatogram no longer overlaps the deionized distilled water dip and can be determined quantitatively. As Cl may be depleted under vacuum by XRF analysis, Cl^−^ is a logical choice to estimate salt concentration. More water-soluble species in salt sources (e.g., sea water; Pytkowicz and Kester 1971) could be measured to reduce the uncertainty.

Depletion of Cl^−^ occurs as H_2_SO_4_ or HNO_3_ reacts with sea salt, which exchanges Cl^−^ for SO_4_^=^ or NO_3_^−^, respectively. This will increase the sea salt mass as SO_4_^=^ (MW = 96) and NO_3_^−^ (MW = 62) are heavier than Cl^−^ (MW = 35) (Bardouki et al. 2003). For coastal samples from Canada, Yao and Zhang (2012) hypothesized Cl^−^ replacement with di-nitrogen pentoxide (N_2_O_5_), instead of HNO_3_, and that SO_4_^=^ may be associated with Cl^−^ depletion under acidic conditions. Sciare et al. (2003) defined sea salt (ss) as the sum of Na^+^, Cl^−^, ssCa^++^, ssK^+^, water-soluble magnesium (Mg^++^), and ssSO_4_^=^; Zhang et al. (2013) substituted ssMg^++^ for Mg^++^, whereas Hsu et al. (2010) used the sum of Na^+^, Cl^−^, and Mg^++^.

### Trace elements

Minor or trace elements, excluding geological species, can be added to the RM. Macias et al. (1981, Eq. 1) summed the trace elements in the form of CuO, ZnO, and PbO. Other studies (i.e., Eqs. 3, 5, 6, and 8) summed remaining elements by XRF, excluding S and the geological elements, with the exception of Solomon et al. (1989, Eq. 2), who also included Na^+^ and Mg^++^. Trace elements are more pronounced in coarse particles or at sampling sites near industrial facilities contaminated with toxic metals (Chow et al. 2002b) when some elements are not accounted for by the mineral formulae in Table 1. More complicated trace element oxides (TEOs; sum of oxides for vanadium (V), manganese (Mn), nickel (Ni), copper (Cu), zinc (Zn), arsenic (As), lead (Pb), selenium (Se), strontium (Sr), phosphorus (P), chromium (Cr), and K) were used by Landis et al. (2001) and Zhang et al. (2013), but this component accounted for a small fraction (0.5–1.6 %) of PM_2.5_ mass. Therefore, summing the remaining elements may be sufficient.

### Others

The remaining mass may be attributed to measurement errors, improper multiplier(s), missing source(s), and/or particle-bound water (e.g., Frank 2006; Malm et al. 2011). This component could represent negative mass if RM overestimates gravimetric mass.

Non-crustal K was estimated as “Others” by Maenhaut et al. (2002), Simon et al. (2011), and Yan et al. (2012) based on either K − 0.6Fe (Eq. 8) or 1.2 × (K − 0.6Fe) (Eq. 11), respectively. Organic acids (sum of acetate, fomite, methane sulfonate, pyruvate, and oxalate) were added to RM by Putaud et al. (2000).

## Applications of mass reconstruction equations to special studies

Supplemental Table S-1 summarizes previous studies which give rise to the 11 RM equations in Table 1. Only a subset of equations (i.e., Eqs. 1, 2, 3, 5, and 8) are applied in these short-term special studies. Concerns over visibility degradation in the southwestern USA prompted the establishment of the Western Fine Particle Network that measured size segregated mass and elements during 1977–1981 (Flocchini et al. 1981). As part of the Denver Winter Haze Study and Project VISTTA, Countess et al. (1980) and Macias et al. (1981) started using RM to determine sources of haze-causing aerosol in uban Denver and non-urban Grand Canyon areas, respectively. Equation 1 was developed by Macias et al. (1981) for PM samples at two remote desert sites near Page, AZ. SO_4_^=^ was not completely neutralized based on the molar ratio of NH_4_^+^ to SO_4_^=^ (1.65 instead of 2.0). RM accounted for 75–93 % of PM_2.5_ and 50–69 % of PM_15–2.5_. Low PM_15–2.5_ RMs were attributed to the absence of carbon measurements.

For nine sites in the SoCAB (Solomon et al. 1989, Eq. 2), RM accounted for 86–94 % (averaging 92 %) of annual PM_10_. Average measured NH_4_^+^ concentrations were 17 % lower than those estimated from (NH_4_)_2_SO_4_ and NH_4_NO_3_, consistent with sulfates being slightly acidic or some of the nitrates being present as NaNO_3_. In another SoCAB study (Chow et al. 1994b, Eq. 3), RM accounted for 70–80 % of PM_2.5_ and 80–85 % of PM_10_ at nine sites during summer; unexplained mass was 5 % lower at six sites during fall. Chow et al. (1994b) measured OC on tandem quartz-fiber filter packs (i.e., OC on quartz-fiber front filter as OC_QF_, followed by a quartz-fiber backup filter as OC_QBQ_) to estimate adsorption of volatile organic compounds (VOCs; Chow et al. 2006a; Subramanian et al. 2004; Turpin et al. 1994), but large variations were found in OC_QBQ_. Average OC field blanks (OC_FB_) are commonly subtracted from OC_QF_ (Chow et al. 2010; Watson et al. 2009). In such cases, RM uses blank subtracted values.

In central California (Chow et al. 1996, Eq. 5), RM accounted for >90 % of PM_2.5_ and PM_10_ at ten sites. At PM concentrations <30 μg/m^3^, the RM often exceeded the measured PM mass. This was in part attributed to OC_QF_ that was not blank-corrected as OC_QBQ_ > OC_QF_ in 168 out of 584 (29 %) samples during ozone episodes. Uncorrected OC_QF_ may be affected by a combination of positive (adsorption) and negative (volatilization) biases (Chow et al. 2010; Watson et al. 2009).

In Melpitz, Germany, RM accounted for 86 % of PM_2_ and 116 % of PM_10–2_ (Maenhaut et al. 2002, Eq. 8). OC was overestimated owing to adsorption of VOCs on quartz-fiber filters, as PM mass was 21 % higher from the quartz-fiber than the collocated Nuclepore-membrane filters. Water associated with hygroscopic species was not accounted for by gravimetry. Considering that the sum of inorganic ions accounted for 34 % of the PM_10–2_, the associated water at 50 % filter equilibration RH could have accounted for the overestimation of PM_10–2_ mass.

## Evaluation of mass reconstruction through analysis of large data sets

Several studies have evaluated RM in the IMPROVE network (see Eqs. 4, 6, 7, and 9–11 in Table 1), the largest and most consistently acquired chemical speciation data set in the world. Malm et al. (1994, Eq. 4) first applied the IMPROVE “soil” formula (Eq. C) to 36 sites, and RM accounted for 75–80 % of PM_2.5_, consistent with an OM underestimation using 1.4OC. Andrews et al. (2000, Eq. 6) reported low RM (58–67 % of PM_2.1_) among four different types of samplers at Great Smoky Mountains National Park. Replacing SO_4_^=^ with (NH_4_)_2_SO_4_ increased RM by 6 %. The corresponding IMPROVE samples yielded RM as 83 % of measured mass. Andrews et al. (2000) attributed the mass deficit to: (1) underestimation of geological minerals; (2) water retention on the Teflon-membrane filter deposit; and (3) underestimation of OM. However, the mineral contribution was too small to account for the deficit. The RM deficiency was reduced to 15–23 % after estimating water content; hygroscopic organics may result in additional particle-bound water (Saxena and Hildemann 1996). In addition to the low OM (1.4OC) estimate, subtracting OC_QBQ_ over-corrected for organic vapor adsorption (Andrews et al. 2000).

Lowenthal and Kumar (2003) applied Eq. 7 to 59 IMPROVE sites from 1988 to 1999. RM averaged 88 %, ranging 61–98 % of PM_2.5_. Incorporating Na, Cl, and trace elements increased RM by 30 % at the coastal Point Reyes site but had a small effect (∼3 %) at other sites. RM accounted for a larger fraction during winter than summer at 51 of 59 sites.

At ∼40 % RH (i.e., IMPROVE filter equilibration conditions for gravimetric analysis), (NH_4_)_2_SO_4_ and NH_4_NO_3_ (Eq. 7) absorb about 0.3 and 0.2 g of water/g of dry compound, respectively, assuming supersaturated (NH_4_)_2_SO_4_ (Chan et al. 1992; Tang and Munkelwitz 1994). The addition of water would increase RM by 11 % in summer and 12 % in winter. A more hygroscopic form of SO_4_^=^ or H_2_SO_4_ is needed during summer to account for the observed seasonal differences. However, this assumption cannot be tested without measured NH_4_^+^ or H^+^ and would not explain the discrepancies when SO_4_^=^ levels are low.

Using 2.1OC (Turpin and Lim 2001) increased RM by 14 % in summer and 16 % in winter (which overestimated measured PM_2.5_). A lower *f* may be applicable in winter due to lower photochemical activity (i.e., less unmeasured O in OM). For IMPROVE sites, monthly median OC_QBQ_ (acquired at ∼5 % of IMPROVE sites) was used for blank subtraction, assuming VOCs adsorbed on both QF and QBQ became saturated (Watson et al. 2009). During 1990–1999, monthly median OC_QBQ_ in summer were 0.155 μg/m^3^ (∼3 % of PM_2.5_) higher than winter. Gaseous organic adsorption and seasonal effects in the OC multiplier, evaluated by Lowenthal and Kumar (2003), narrowed the seasonal RM deficit.

PM_2.5_ sampling methods in both the IMPROVE network and CSN result in artifacts for RM (DeBell et al. 2006, Eq. 9; Hand et al. 2011, Eq. 10). Malm et al. (2011) addressed the uncertainties in PM_2.5_ gravimetric and speciation measurements. PM_2.5_ ions (e.g., Cl^−^, NO_3_^−^, and SO_4_^=^) are measured on a nylon-membrane filter after a denuder to remove HNO_3_, which captures both non-volatilized and volatilized NO_3_^−^. Particulate NH_4_NO_3_ exists in equilibrium with gaseous HNO_3_ and ammonia (NH_3_) (Hering and Cass 1999) depending on temperature, pressure, and RH. During sampling, NO_3_^−^ can evaporate as HNO_3_ due to the pressure drop across the filter and be re-absorbed as volatilized NO_3_^−^. However, volatilized NO_3_^−^ is not part of the gravimetric mass, resulting in a negative artifact, which is most prominent during summer. The uptake of water by sulfates, nitrates, and organics during weighing (at ∼40 % RH) counterbalances NO_3_^−^ volatilization from the Teflon-membrane filter (Chow et al. 2005).

Blank subtraction is applied to OC_QF_ for IMPROVE samples but not for CSN samples (Chow et al. 2010; Watson et al. 2009). For the period prior to 2007/2008, carbon analysis followed the STN_TOT protocol in CSN (thermal/optical transmittance; Peterson and Richards 2002) and the IMPROVE_TOR protocol in IMPROVE (thermal/optical reflectance; Chow et al. 1993). Although total carbon (TC = OC + EC) is comparable, STN_TOT reports higher OC and lower EC than the IMPROVE_A_TOR protocol (Chow et al. 2007c). Malm et al. (2011) used collocated measurements in order to relate CSN to IMPROVE carbon concentrations using ordinary least squares (OLS; unweighted) regression:

D $$ {\mathrm{PM}}_{2.5} = \mathrm{a}1 \times 1.375\ {\mathrm{SO}}_4^{=} + \mathrm{a}2 \times 1.29\ {\mathrm{NO}}_3^{-} + \mathrm{a}3 \times \mathrm{O}\mathrm{C} + \mathrm{a}4 \times \mathrm{O}\mathrm{ther} $$

where “Other” is the sum of EC, geological minerals, and salt (DeBell et al. 2006, Eq. 9). The two regression coefficients, a1 and a2, should equal unity if SO_4_^=^ and NO_3_^−^ are present as (NH_4_)_2_SO_4_ and NH_4_NO_3_, respectively. Equation D assumes no water uptake at weighing equilibration conditions and no NH_4_NO_3_ evaporation during sampling. a3 is the OC multiplier (*f*) and a4 = 1 if the weighting factors for geological minerals and salt are correct. For 168 IMPROVE sites during 1988–2008, average a1, a2, a3, and a4 values were 1.12, 0.75, 1.60, and 1.06, respectively. This implies a 12 % contribution from water mass associated with (NH_4_)_2_SO_4_ during weighing, a net loss of 25 % NH_4_NO_3_ during sampling, and an OC multiplier of 1.6 with 6 % more EC, geological minerals, and salt. A higher a3 for OC was found during summer (*f* = 1.7) than winter (*f* = 1.42), with a lower a2 during summer showing more NH_4_NO_3_ evaporation, as expected.

Different regression analyses were conducted for 708 IMPROVE samples at the urban Fresno Supersite (Watson et al. 2000) from 2004 to 2010, as shown in Table 2. Ordinary weighted least squares (OWLS) regression takes into account the measurement uncertainty of the independent variable (i.e., PM_2.5_), while effective variance (EV) regression takes into account the uncertainties of both the independent and dependent variables and should provide the most realistic results. Table 2 shows that average PM_2.5_ NO_3_^−^ (3.9 ± 4.9 μg/m^3^) and OC (3.2 ± 2.5 μg/m^3^) were the major components, with 1.33 ± 1.26 μg/m^3^ for SO_4_^=^. The average EC, geological minerals, and salt concentrations were 0.93, 1.42, and 0.27 μg/m^3^, respectively. Without accounting for measurement uncertainties, a large OLS a1 of 1.61 for SO_4_^=^ yields an increment (1.61–1.00 = 0.61) five times higher than the 0.12 increment (i.e., regression coefficient of 1.12) from Malm et al. (2011)—this is inconsistent with 30–40 % RH weighing conditions. The 8 % NO_3_^−^ volatilization (i.e., a2 = 0.92) and an OC multiplier (a3) of 1.67 in Table 2 seem reasonable for typical ion concentrations. The geological mineral mass is overestimated (a4 = 0.59) by the IMPROVE “soil” formula (Eq. C).

**Table 2:** Regression coefficients for mass reconstruction (Eq. D) using various regression methods for Interagency Monitoring of Protected Visual Environments (IMPROVE) network samples collected at urban Fresno supersite in CA from 3 September 2004 to 31 December 2010

	OLS^b^	OWLS^c^	EV^d^	Average ± standard deviation^e^	Minimum–maximum
Category^a^					
Coefficient a1 (SO_4_^=^)	1.61	0.90	0.93		
Coefficient a2 (NO_3_^−^)	0.92	0.85	0.88		
Coefficient a3 (OC)	1.67	1.74	1.71		
Coefficient a4 (Other)	0.59	0.78	0.78		
Species					
Avg. SO_4_^=^ (μg/m^3^)				1.33 ± 1.26	0.079–25
Avg. NO_3_^−^ (μg/m^3^)				3.9 ± 4.9	0.138–38
Avg. OC (μg/m^3^)				3.2 ± 2.5	0.54–24
Avg. Other (μ/m^3^)				2.6 ± 1.8	0.53–26

^a^ http://views.cira.colostate.edu/web/. To ensure data quality, only samples with species concentrations exceeding their uncertainties were included for regression analyses

^b^Ordinary least squares − no weighting

^c^Ordinary weighted least squares − weighting depends on uncertainty of independent variable

^d^Effective variance least squares − weighting depends on uncertainties of both the independent (i.e., SO_4_^=^, NO_3_^−^, OC, and Other) and dependent variables (Watson et al. 1984)

^e^Average and calculated ranges are as follows (number of samples in all averages = 708)

Table 2 shows a1 < 1 (0.90–0.93) by OWLS and EV regression methods, implying SO_4_^=^ is somewhat acidic in Fresno, which is probably not the case. NH_3_ is abundant in this agricultural region (e.g., Chow et al. 1998, 1999, 2006b). The a2 of 0.85–0.88 is slightly lower than 0.92 in OLS, but it is consistent with NO_3_^−^ volatilization. The a3 is 2–4 % higher in OWLS (1.74) and EV (1.71) than OLS (1.67), but a4 (0.78) is ∼30 % higher than OLS (0.59). The high a1 and low a4 in the OLS regression are not realistic. However, the regressions in all cases are statistically significant and the squared multiple correlations (*r* ^2^) are 0.98 or 0.99. Hand et al. (2011) and Malm et al. (2011) provide insights into sampling and analytical artifacts in long-term PM_2.5_ networks. However, the example illustrated for Fresno indicates limitations on generalizing from a single dataset and one statistical approach.

Simon et al. (2011, Eq. 11) employed data screening procedures to eliminate suspect or physically unreasonable concentrations. Data sets with correlation coefficients (*r*) among explanatory variables greater than the absolute value of 0.85 were eliminated; whereas EC was removed due to correlations with OC. However, the effects of collinearity fell along a continuum, and selecting the level of correlation that can be tolerated is subjective. OLS regression was found to produce more bias in regression coefficients than OWLS or EV. Overall, the estimated median OC multipliers (a3) at the 50th percentile were lower for winter (*f* = 1.39) and fall (*f* = 1.59) and comparable between spring (*f* = 1.83) and summer (*f* = 1.81). The lowest median a3 (*f* = 1.29) was estimated at western sites during winter. Simon et al. (2011) concluded that more realistic and unbiased estimates of the OC multiplier were obtained using an “error in variables” regression and eliminating EC.

## Major factors influencing mass reconstruction

The key factors affecting RM are examined for: (1) the OC multiplier (*f*); (2) sampling artifacts; (3) carbon analysis methods; (4) ammonium and nitrate volatilization; and (5) water uptake on Teflon-membrane filter deposits at different equilibration RHs.

### Measurement of the OC multiplier (*f*) to estimate OM

Several aerosol extraction (a combination of water, organic solvents, and/or solid-phase extraction) and analytical methods (e.g., elemental analysis, Fourier-transform infrared (FTIR) spectroscopy, quadrupole-aerosol mass spectrometer (Q-AMS), etc.) have been applied to estimate the *f* multiplier (i.e., the OM/OC ratio). As shown in Table 3, the results from these direct measurements are variable with *f* = 1.27–2.2. Aircraft sampling with FTIR often yielded *f* = ∼1.3–1.4 (Gilardoni et al. 2007; Maria et al. 2002; Russell 2003) with a higher *f* multiplier (1.6–1.8) found by Takahama et al. (2011). Lower *f* values (∼1.4) were also found for personal and indoor sampling (Reff et al. 2007), for ship emissions (∼1.6 by Gilardoni et al. 2007), and for urban areas (∼1.6 by Day et al. 2010; Hawkins and Russell 2010; Ruthenburg et al. 2014). Higher *f* values (∼2.0 to 2.2) were typically found for aged aerosols sampled in remote areas (e.g., Gilardoni et al. 2007; Takahama et al. 2011).

**Table 3:** Examples of OM/OC ratio determined in various studies at urban and remote locations

Study	Particle size	Method/description^a^	OM/OC (ratio)		Location	Season (sampling period)
			Urban/sub-urban	Remote		
Krivacsy et al. (2001)	PM_2.5_	Used total organic carbon (TOC) analyzer to determine TC and WSOC		1.9	High alpine research station, Jungfraujoch, Switzerland (in the Swiss alps; elevation 3580 m above sea level (asl))	July to August 1998
		Used solid-phase extraction on a copolymer sorbent				
		Analyzed C, H, N, and S of OM by elemental analyzer with estimated O				
		Determined OM mass by gravimetry				
Kisset al. (2002)	PM_1.5_	Used total organic carbon (TOC) analyzer to determine TC and WSOC		1.93 ± 0.038 (ranged from 1.9 to 2.0)	Rural K-puszta site with mixed forest, Hungary	January to September 2000
		Used solid-phase extraction on a copolymer sorbent				
		Analyzed C, H, N, and S of OM by elemental analyzer with estimated O				
		Determined OM mass by gravimetry				
Maria et al. (2002, 2003)	PM_1_	Calculated OC and OM from FTIR and compare with thermal/optical OC		1.27 ± 0.02 to 1.49 ± 0.28	Aircraft sampling over northeast Asia during the ACE-Asia Campaign	April and May 2001
		A 4-solvent rinsing procedure was used to separate functional groups into fractions of increasing hygroscopicity				
		Used carbon monoxide (CO) vs. FTIR OC ratios to classify back trajectory clusters into 10 groups				
Russell (2003)	Submicron PM	FTIR, estimated OC from the number of carbon bonds and OM from the molecular mass of each functional group		1.36 ± 0.13 (1.2–1.6)	Aircraft and ship-based sampling in the Caribbean and northeastern Asia^b^	March to April and July 2001
El-Zanan et al. (2005)	PM_2.5_	After sequential solvent extraction with dichloromethane, acetone, and water, the dried residue was weighed for OM and analyzed for OC by TOR OC. The water extracts were also analyzed for ions (Cl^-^, NO_3_^-^, SO_4_^=^, Na^+^, K^+^, and NH_4_^+^) to subtract inorganic ion mass.		1.92 ± 0.40 (1.58–2.58) 2.07 by mass balance	U.S. National Parks (5 sites)^c^	Annual (1988–2003)
Zhang et al. (2005)	PM_1_	Inorganic ions (e.g., sulfates, nitrates, ammonium) and organics by AMS, followed by deconvolution of AMS mass spectrum to identify HOAs and OOAs.	Averaged 1.8 with 1.2 for HOA and 2.2 for OOA		Pittsburgh, PA	September 2002
Yu et al. (2005a, b)	PM_1.5_	Used water and solvent extraction followed by GC/MS analysis for WSOC and solvent-soluble OC		Daytime 2.0 ± 0.3 (1.4–2.5). Nighttime 1.8 ± 0.2 (1.3–2.0)	Great Smoky Mountains National Park, TN	July to August 2005
Chen and Yu (2007)	PM_2.5_	Determined OM by combining heating, gravimetric, and chemical constituents	2.1 ± 0.3		Sub-urban site at Clearwater, Hong Kong	October 2003 to June 2005
Gilardoni et al. (2007)	PM_1_	FTIR and comparison with IC-PILS for speciated carboxylic acids		1.4 ± 0.12	Aircraft sampling of Ohio power plant emissions and regional background (12 flights)	Summer 2004
				1.6 ± 0.4	Ship sampling in the Gulf of Maine	
				1.5 ± 0.16	Appldore Island, ME	
				1.6 ± 0.14	Chebogue Point, Nova Scotia, Canada	
Reff et al. (2007)	PM_2.5_	FTIR for aliphatic (CH) and carbonyl (C=O and [(C=O)−OH] by partial least squares (PLS) equation	Outdoor 1.7–2.6		219 non-smoking homes in LA county, CA, Elizabeth, NJ, and Houston, TX	Summer 1999 to Spring 2001
			Indoor 1.3–1.7 (average 1.45 ± 0.17)			
			Personal 1.3–1.6 (average 1.4 ± 0.11)			
Aiken et al. (2008)	PM_1_	Elemental analysis by AMS	Average 1.71 with 1.2–1.3 for HOA, 1.85–2.45 for OOA; and 1.6–1.7 for BBOA		Mexico City, Mexico^d^	March 2006
Cozic et al. (2008)	PM_1_	OM by Q-AMS, normalized to OC by OC/EC TOT carbon analyzer		1.84	Jungfraujoch, Switzerland	February and March 2005
Polidori et al. (2008)	PM_2.5_	Used a combination of polarity-based extraction/fractionation method, determine OM by gravimetry and OC by thermal/optical analysis (polarity generally increases as organic oxygen content increases)	OM/OC ratios increase with increasing polarity: 1.37 for hexane, 1.66 for dichloromethane, 1.89 for ethyl acetate, 2.11 for acetone, and 2.25 for methanol extractions. Annual average ratios with (OM/OC_total_) and without (OM/OC_extract_) non-extractable material were 2.05 ± 0.18 and 1.91 ± 0.24, respectively		Pittsburgh, PA	Annual (July 2001–July 2002)
Gilardoni et al. (2009)	PM_1_	FTIR	1.8		Mexico City, Mexico	March 2006
				2.0	Altzomoni (60 km SE of Mexico City, Mexico)	
Day et al. (2010)	PM_1_	FTIR and comparison of OM with Q-AMS	1.66^e^		La Jolla, CA	February and March 2009
Hawkins and Russell (2010)	PM_1_	FTIR and comparison with Q-AMS	1.55 ± 0.17		La Jolla, CA	June to September 2008
Takahama et al. (2011)	Submicron PM	FTIR and comparison with ACSM		2.0–2.2	Whistler Mountain, BC, Canada	March and April 2009
				1.6–1.8	Aircraft sampling over Mexico and the Gulf of Mexico coast (12 flights)	May to September 2009
Ruthenburg et al. (2014)	PM_2.5_	FTIR		1.83	Mesa Verde, CO	Annual (2011) at seven IMPROVE sites
				1.79	Olympia, WA	
				1.78	Proctor Maple R.F., VT	
				1.71	St. Marks, FL	
				1.73	Trapper Creek, AK	
			1.56		Phoenix, AZ	

*PM* particulate matter, *PM*_*x*_ PM with diameter smaller than *x* micrometers at 50 % cut-point, *HOA* hydrocarbon-like organic aerosol (represent gasoline and diesel engine exhaust emissions), *OOA* oxygenated organic aerosol (contains more oxygen atoms than HOAs, resemble humic-like substance, and have been associated with secondary organic aerosol), *BBOA* biomass burning organic aerosol

^a^Measurement methods include aerosol chemical speciation monitor (ACSM), aerodyne aerosol mass spectrometer (AMS), quadrupole-aerosol mass spectrometer (Q-AMS), ion chromatography-particle into liquid sampler (IC-PILS), Fourier transform infrared analysis (FTIR), total carbon (TC), thermal/optical reflectance (TOR), thermal/optical transmittance (TOT), water-soluble organic carbon (WSOC), water-soluble organic matter (WSOM)

^b^During the aerosol characterization experiment (ACE)-Asia study in the western Pacific and the Passing Efficiency of the Low Turbulence Inlet Experiment (PELTI) study in the Caribbean

^c^Sites are Acadia, ME; Great Smoky Mountains, TN; Big Bend, TN; Indian Gardens, Grand Canyon, AZ; and Mount Rainier, WA

^d^During the Megacity Initiative: Local and Global Research Observations (MILAGRO) field campaign, ground-based sampling was done at the T0 Supersite at the Instituto Mexicano del Petróleo (IMP) and aircraft data were collected aboard the NCAR C-130 aircraft over the city

^e^Estimated based on the sum of carbon mass in the functional groups (Russel 2003)

Weighing samples before and after solvent extraction (Japar et al. 1984) resulted in *f* = 1.4 for diesel exhaust samples. In Pittsburgh, PA, Polidori et al. (2008) found that *f* increased with increasing polarity with *f* higher in summer (June and July) and winter (December and January) than in spring (March) and fall (October and November). High summer and winter values (*f* = 2.08–2.11) were attributed to biomass burning and residential wood combustion (RWC), respectively. Accounting for both solvent extractable and non-extractable material, the annual average *f* was estimated to be 2.05 ± 0.18.

Based on AMS measurements and multivariate analyses (e.g., principle component analysis (PCA), regression analysis, and positive matrix factorization (PMF)), Zhang et al. (2005) and Aiken et al (2008) reported average *f* = 1.7–1.8 with *f* = 1.2–1.3 for hydrocarbon-like organic aerosols (HOAs) and *f* = 1.9–2.5 for oxygenated OA (OOA). Aiken et al. (2008) also reported *f* = 1.6–1.7 for biomass burning OA (BBOA). Based on a series of field studies, Philip et al. (2014) parameterize OM/OC from AMS measurements using *f* = 1.3 for primary organic aerosol and *f* = 2.1 for OOA. The OM/OC ratio is determined as 1.3(*f*_POA_) + 2.1(1–*f*_POA_), where *f*_POA_ is the primary organic aerosol (POA) fraction of the AMS data, a proxy for combustion emissions (derived from ambient NO_2_ measurements). The OM/OC ratios ranged from 1.7 to 2.1.

The *f* multiplier is expected to be higher in rural than in urban areas due to oxidation and/or addition of SOA during transport. However, the results in Table 3 do not show systematic variations. Organic compounds vary by location, season, and time of day. Site-specific *f* values need to be measured.

### Sampling and analysis artifacts

Different approaches to sampling and analysis introduce uncertainties and systematic biases, including carbon sampling artifacts, thermally evolved carbon analysis methods, ammonium and nitrate volatilization, and particle-bound water on Teflon-membrane filters. The following subsections address these measurement uncertainties.

#### Carbon sampling artifacts and carbon analysis by thermal evolution

As noted, PM_2.5_ sampling onto quartz-fiber filters is accompanied by positive (e.g., VOC adsorption) and negative (e.g., volatilization during and after sample collection) OC artifacts (Chow et al. 2010; Putaud et al. 2000; Turpin et al. 1994; Watson et al. 2009). Positive artifacts (e.g., estimated by field blank (OC_FB_), backup filter (OC_QBQ_), preceding organic denuders, and regression analyses) often exceed negative artifacts (ten Brink 2004; Watson et al. 2009). OC artifacts may bias EC values by as much as ∼50 %, especially by TOT, as light attenuation due to charring of the adsorbed organics within the filter has greater influence than charring of the surface particle deposit in TOR (Chen et al. 2004; Chow et al. 2004).

In a review of carbon comparison studies, Watson et al. (2005) found EC differed by up to a factor of seven (Schmidt et al. 2001) among 19 thermal evolution methods. Table 4 summarizes the three most widely applied thermal/optical carbon analysis protocols (i.e., IMPROVE_A_TOR, STN_TOT, and EUSAAR_2_TOT). The US long-term networks (e.g., IMPROVE and CSN) apply the IMPROVE_A_TOR protocol (USEPA 2006). The European Union EUSAAR-2 protocol (Cavalli et al. 2010; Panteliadis et al. 2015) is similar to the IMPROVE_A temperature protocol with variations in selected temperature plateaus and shorter (70–180 s) residence times. Higher OC values in TOT can result in lower OM/OC ratios and might bias RM.

**Table 4:** Comparison of common thermal/optical protocols: IMPROVE_A, STN, and EUSAAR_2

Carbon fraction	Atmosphere^d^	IMPROVE_A_TOR^a^		STN_TOT^b^		EUSAAR_2_TOT^c^	
		Temp. (°C)	Residence time (s)^e^	Temp. (°C)	Residence time (s)	Temp. (°C)	Residence time (s)
OC1	Inert	140	80–580	310	60	200	120
OC2	Inert	280	80–580	480	60	300	150
OC3	Inert	480	80–580	615	60	450	180
OC4	Inert	580	80–580	900	90	650	180
	Oven cooling^f^	NA	NA	NA	30	NA	30
EC1	Oxidizing	580	80–580	600	45	500	120
EC2	Oxidizing	740	80–580	675	45	550	120
EC3	Oxidizing	840	80–580	750	45	700	70
EC4	Oxidizing	NA	NA	825	45	850	80
EC5	Oxidizing	NA	NA	920	120	NA	NA

*NA* not applicable

^a^The non-urban Interagency Monitoring of Protected Visual Environments (IMPROVE) network and urban Chemical Speciation Network (CSN), measures and reports both thermal/optical reflectance (TOR), and thermal/optical transmittance (TOT), following the IMPROVE_A_TOR protocol (Chow et al. 2007b, 2011)

^b^Speciation Trends Network (STN), also called NIOSH-like protocol (Peterson and Richards 2002)

^c^European Supersites for Atmospheric Aerosol Research, EUSAAR_2, protocol (Cavalli et al. 2010)

^d^Inert atmosphere ultra-high purity (UHP) helium (He) for OC analysis. Oxidizing atmosphere 98 % He/2 % oxygen (O_2_) for all protocols

^e^Ramping to the next temperature or atmosphere begins when the flame ionization detector (FID) response returns to either baseline or a constant value; these times represent minimum and maximum times to be spent in any segment, respectively

^f^At the end of OC analysis, a cooling blower turns on for ∼30 s. EC analysis starts ∼10 s after the introduction of 98 % He/2 % O_2_

#### Ammonium and nitrate volatilization

Compared with total particulate NO_3_^−^, Chow et al. (2005) found volatilized NO_3_^−^ losses ranging from <10 % during cold months to >80 % during warm months (from the front quartz-fiber filter) for urban and non-urban sites. The amount of NH_4_NO_3_ volatilization from the Teflon-membrane filter can be estimated by a thermodynamic model (Hering and Cass 1999; Mozurkewich 1993), but this is only possible when gaseous HNO_3_ and NH_3_, total particle NO_3_^−^, temperature, and RH are known (Chow et al. 2005; Stelson et al. 1979). Volatilized NO_3_^−^ is not considered in the USEPA’s (1997) PM_2.5_ Federal Reference Method (FRM) for compliance monitoring. However, for evaluating light extinction or health effects, it is necessary to account for NO_3_^−^ volatilization during sampling.

Yu et al. (2005c) noted that gaseous HNO_3_ interacts with nylon filters and retains HNO_3_ that volatilized from NH_4_NO_3_. However, losses of NH_4_^+^ (i.e., gaseous NH_3_) from nylon filters after a Na_2_CO_3_ denuder for the selected six IMPROVE sites ranged from 10 to 28 % (monthly average) during summer. Yu et al (2006) found that, for individual samples, the NH_4_^+^ losses spread between 1 and 65 %. NH_4_^+^ volatilization is enhanced by increasing temperature and RH, and with the fraction of total NH_*x*_ (sum of NH_3_ and NH_4_^+^) present as NH_3_ (Chen et al. 2014).

Losses of NH_4_^+^ after sampling need to be investigated. Non-volatilized NH_4_^+^ can be acquired on Teflon-membrane or quartz-fiber filters without preceding denuders. Ideally, both non-volatilized and volatilized NH_4_^+^ should be acquired on a parallel channel, using a preceding citric acid denuder to remove NH_3_, followed by a quartz-fiber filter with a citric acid impregnated cellulose-fiber backup filter (e.g., Chow 1995; Chow et al. 1998).

#### Particle-bound water on the Teflon-membrane filter

The influence of particle-bound and particle-adsorbed water on PM has been explored in several studies (e.g., Frank 2006; Malm et al. 2011; Malm and Day 2001; Perrino et al. 2013; Rees et al. 2004; Temesi et al. 2001). Water associated with PM was estimated by Harrison et al. (2003) by applying 1.29 to the sum of (NH_4_)_2_SO_4_ and NH_4_NO_3_ concentrations and in others (e.g., Murillo et al. 2012; Siddique and Waheed 2014) by multiplying 0.32 to the sum of NH_4_^+^ and SO_4_^=^.

Hygroscopic salts (e.g., (NH_4_)_2_SO_4_, NH_4_NO_3_, and NaCl) absorb water as a function of RH (Chan et al. 1992; Tang and Munkelwitz 1994). At the deliquescence RH (DRH; ∼80 %), dry (NH_4_)_2_SO_4_ particles start to absorb water and the amount rises with increasing RH. The hydrated particle retains water below the DRH until it re-crystallizes at the efflorescence RH (ERH) of ∼30–40 %, the hysteresis effect (e.g., Han and Martin 1999). Acidic H_2_SO_4_ absorbs and desorbs water continuously with changes in RH, without exhibiting deliquescence or efflorescence. The DRH and ERH of pure NH_4_NO_3_ are 62 and 32 %, respectively. Tang et al. (1997) found that sea salt begins to deliquesce at low RH in the presence of Mg^++^ and Ca^++^, but that most of the material deliquesces between 70 and 74 %, the DRH of NaCl. Day et al. (2000) and Malm et al. (2003) found little evidence for deliquescence or efflorescence in ambient aerosols at the IMPROVE sites.

At RH >80 %, water may constitute more than 50 % of PM_2.5_ mass (Chen et al. 2003; McMurry 2000). If particles were hydrated during sample collection, the sample filters may retain water for weighing (equilibration of RH 30–40 %; USEPA 1997), unless they were dried below ERH between sample collection and weighing. Based on theoretical thermaldynamic modeling of salt mixtures, Pilinis et al. (1989) found that aerosol may contain up to 30 % water for RH = ∼20–50 %. McInnes et al. (1996) observed that water associated with sea salt particles contributed 26 % of the mass at 40 % RH. Speer et al. (2003) measured changes in PM_2.5_ mass as a function of RH in a humidity-controlled chamber (increased from 4 to 94 % in 5 % increments and then decreased similarly to 12 %) using a beta attenuation monitor (BAM) on Teflon-membrane filters. For samples collected at Research Triangle Park, NC, Speer et al (2003) observed hysteresis in most cases.

The water-soluble organic carbon (WSOC) portion of OM can enhance or inhibit water absorption by inorganic salts (Facchini et al. 1999; Mircea et al. 2002; Saxena et al. 1995; Saxena and Hildemann 1997). At Great Smoky Mountains National Park during the summer of 2006, Lowenthal et al. (2009) reported the water uptake as 5 % PM_2.5_ WSOC at 45 % RH and 33 % at 80 % RH. Based on thermodynamic modeling (Chen et al. 2003; Clegg et al. 1998; Tang and Munkelwitz 1994), ∼80 % of the measured water can be associated with SO_4_^=^ and NO_3_^−^. Speer et al. (2003) attributed the ∼20 % “residual water” to organics; the amount of water per unit mass of organics was ∼50 % of that associated with (NH_4_)_2_SO_4_ (per unit mass) at 60–80 % RH. Conversely, Engelhart et al. (2011) determined that water growth of aerosols in Crete, Greece, was consistent with thermodynamic modeling based on inorganic constituents alone. Water mass on the Teflon-membrane filter can be determined by weighing the filter under equilibrium conditions (30–40 % RH), drying the filter completely in a desiccator, and then rapidly re-weighing.

Recent advances in thermodynamic models have incorporated some organic compounds to estimate the associated water activity (Clegg et al. 2001, 2008; Clegg and Seinfeld 2006). However, most of the organic species have not been identified, and their thermodynamic properties are uncertain (Saxena and Hildemann 1996; Sempéré and Kawamura 1994). While thermodynamic modeling may provide insights on particle-bound water, the most straightforward means is through direct gravimetric analysis over a range of RHs.

## Summary and conclusions

As PM_2.5_ mass concentration has been regulated in NAAQS to protect public health and welfare, it is important to understand the particle composition in order to: (1) examine the causes of elevated concentrations; (2) attribute ambient concentrations to air pollution sources; (3) relate toxic components to public health and ecosystems; and (4) associate particle scattering and absorption properties with visibility impairment, the Earth’s radiation balance, and climate change. With advances in sampling and analysis techniques, the demand for characterizing the chemical, physical, and optical properties of atmospheric aerosol is increasing worldwide. The validity of mass and chemical measurements needs to be examined prior to or in conjunction with air-quality modeling to develop pollution control strategies and reduce human exposure to hazardous pollutants.

Mass reconstruction is a simple and useful tool for validating the consistencies and addressing uncertainties among mass and chemical measurements. The reconstruction of measured mass was started by Countess et al. (1980) and Macias et al. (1981) as PM chemical speciation for ions, carbon, and elements became available. The 11 reconstructed mass (RM) equations examined here provide history and insight into the evolution of RM. Major PM components include: (1) major inorganic ions (e.g., SO_4_^=^, NO_3_^−^, and NH_4_^+^); (2) OC and its multiplier (*f*) to estimate OM, (3) EC, (4) geological minerals (based on estimated metal oxides), (5) salt, (6) trace elements (excluding double counting of ions and crustal components in geological minerals), and (7) others (as remaining mass including particle-bound water). The remaining mass can be negative when RM overestimates the gravimetric mass.

For inorganic ions, either the sum of (NH_4_)_2_SO_4_ and NH_4_NO_3_ (calculated by their respective stoichiometric multiplier as 1.375SO_4_ and 1.29NO_3_^−^) or the sum of SO_4_^=^, NO_3_^−^, and NH_4_^+^ is most commonly applied. For coastal environments, variations account for non-sea salt SO_4_^=^ (nssSO_4_), CaSO_4_, Na(NO_3_)_2_, and NH_4_Cl. The assumption that SO_4_^=^ is completely neutralized as (NH_4_)_2_SO_4_ overestimates SO_4_^=^ mass when non-neutralized (acidic) sulfates are present. Summing of SO_4_^=^, NO_3_^−^, and NH_4_^+^ will not account for H associated with partially neutralized SO_4_^=^ (e.g., NH_4_HSO_4_). Ion balances should be applied to ensure the molar equivalence between the measured anions and cations and to justify the degree of neutralization. NH_4_^+^ measurements should be included in routine monitoring networks and special studies, preferably on a quartz-fiber filter or with preceding citric acid denuder and citric acid impregnated backup filter that can capture both non-volatilized and volatilized NH_4_^+^, respectively.

PM_2.5_ NH_4_NO_3_ may evaporate from Teflon-membrane and quartz-fiber filters during warm, non-winter periods, but its contribution to RM is expected to be highest during winter when low temperatures and high RH favor the particle phase. Ammonium and nitrate volatilization during sampling does not affect mass reconstruction. However, positive bias in RM is expected for CSN and the IMPROVE network where total particulate NO_3_^−^ measured on a nylon-membrane filter includes volatilized NO_3_^−^ that is not part of the gravimetric mass on Teflon-membrane filters. To account for this bias, gaseous HNO_3_ can be removed with a preceding denuder and volatilized NO_3_^−^ can be collected on a nylon filter or salt-impregnated filter behind one of the filters.

The OC multiplier (*f*) ranges from 1.2 to 2.6, depending on the extent of OM oxidation. The most commonly applied multipliers are 1.4 for urban and 1.8 for non-urban sites. The *f* multiplier is expected to be highest in non-urban areas due to oxidation and/or addition of secondary organic compounds during transport. Organic compounds vary by location, season, and time of day. Site-specific *f* values need to be measured. Future studies should focus on direct measurement of the OM/OC ratio at urban and remote locations with sampling periods covering warm and cold seasons.

Organic sampling artifacts need to be quantified using preceding carbon denuders, field blanks, and/or backup filters. Subtracting averaged field blanks from OC is the most convenient way to remove passive organic adsorption. Different thermal/optical carbon analysis protocols may result in additional uncertainties. The analysis protocol used in the CSN prior to 2007/2008 overestimated OC and consistently led to high-biased RM. Consistent carbon analysis protocol should be applied nationwide and internationally. Among the seven PM_2.5_ components, EC is the most straightforward as a single component without any multiplier. However, the abundance of EC is method dependent as OC and EC are operationally defined.

For geological minerals containing Al, Si, Ca, and Fe, compounds are assumed to be Al_2_O_3_, SiO_2_, CaO, and Fe_2_O_3_, respectively, with variations including or excluding FeO, K_2_O, and TiO_2_. The IMPROVE “soil” formula applies a factor of 1.16 to account for unmeasured compounds and tends to overestimate geological minerals. This can be examined empirically by measuring the chemical composition of local geological samples after subtracting OM and ionic concentrations. Since geological minerals are not a major component of PM_2.5_, variations in the assumptions regarding metal oxides or multipliers do not contribute to large variations in RM, but they are important for PM_10–2.5_ and PM_10_ RMs. Trace elements as a sum of remaining elements by XRF (excluding S and geological elements) or as complicated trace element oxides only account for a small fraction (0.5–1.6 %) of PM_2.5_ mass.

There is no standard method to estimate salt. It is mainly based on: (1) the sum of elements (excluding Cl and Cl^−^) to Na or Na^+^ ratio in seawater; (2) straight sum of Na^+^ and Cl^−^; or (3) estimated as 1.8Cl^−^ as in the revised IMPROVE equation. Depletion of Cl^−^ by reaction with sea salt particles with a strong acid (e.g., H_2_SO_4_ and HNO_3_) is difficult to estimate without additional measurement. However, the salt component should be accounted for at sampling sites near coastal areas, salt lakes, or desert playas, as it may comprise up to 20–30 % of PM_2.5_ mass.

Potential bias in measured mass due to the absorption of water by hygroscopic species on the Teflon-membrane filter from which PM_2.5_ mass is determined can be estimated theoretically from concentrations of water-soluble species measured on nylon-membrane or quartz-fiber filters using a thermodynamic model.

In conclusion, the principal sources of uncertainty are: (1) ammonium and nitrate volatilization and inconsistency between total particulate NO_3_^−^ on nylon-membrane filters and non-volatilized NO_3_^−^ on Teflon-membrane filters; (2) unknown OC multipliers (*f*) to estimate OM; (3) inaccurately accounting for OC sampling artifacts; (4) differences among OC and EC analytical protocols; (5) inaccurate conversion of crustal element concentrations to geological minerals; (6) various degrees of Cl^−^ depletion at coastal locations; and (7) particle-bound water on the Teflon-membrane filter deposits. Reasonably accurate PM_2.5_ mass reconstruction can be accomplished by minimizing sampling artifacts and conducting comprehensive chemical analyses to ensure mass closure.

## Electronic supplementary material

Below is the link to the electronic supplementary material.

ESM 1 (DOCX 89 kb)
