# Supplementary material for: Mass reconstruction methods for PM2.5: a review
Source: Air Qual Atmos Health. 2015 May 7;8(3):243–63. doi: 10.1007/s11869-015-0338-3 (PMC4449935; doi:10.1007/s11869-015-0338-3)
Supplement: Supplementary file 1 — (DOCX 89 kb) [file 11869_2015_338_MOESM1_ESM.docx]

**Supplemental Information**

**Mass Reconstruction Methods for PM_2.5_: A Review**

**Judith C. Chow^1,2,3*^, Douglas H. Lowenthal^1,3^, L.-W. Antony Chen^1,4^, Xiaoliang Wang^1,3^, John G. Watson^1,2,3^**

*^1^Desert Research Institute, Reno, Nevada 89512, USA*

*^2^The State Key Laboratory of Loess and Quaternary Geology, Institute of Earth Environment, Chinese Academy of Sciences, Xi’an, Shaanxi, 710075, China*

*^3^Graduate Faculty, University of Nevada, Reno, Nevada 89503, USA*

*^4^Department of Environmental and Occupational Health, University of Nevada, Las Vegas 89154, USA*

^*^Corresponding author. Tel.: +1 775 674 7050; fax: +1 775 674 7009; email address: [Judith.Chow@dri.edu](mailto:Judith.Chow@dri.edu)

Table S-1 Summarizes the approach and results of recent studies applying different reconstructed mass (RM) methods to chemically-speciated particulate matter (PM) measurements.

# References

Andrews E, Saxena P, Musarra S, Hildemann LM, Koutrakis P, McMurry PH, Olmez I, White WH (2000) Concentration and composition of atmospheric aerosols from the 1995 SEAVS Experiment and a review of the closure between chemical and gravimetric measurements. J. Air Waste Manage. Assoc. 50:648-664

Birch ME, Cary RA (1996) Elemental carbon-based method for monitoring occupational exposures to particulate diesel exhaust. Aerosol Sci. Technol. 25:221-241

Chow JC, Watson JG, Lowenthal DH, Pritchett LC, Richards LW (1990) San Joaquin Valley Air Quality Study, Phase 2: PM_10_ modeling and analysis, Volume I: Receptor modeling source apportionment. DRI 8929.1F Desert Research Institute, Reno, NV

Chow JC, Watson JG, Lowenthal DH, Lu Z, Frazier CA, Pritchett LC, Hinsvark BA (1992) San Joaquin Valley Air Quality Study (SJVAQS)/Atmospheric Utility Signatures - Predictions and Experiment (AUSPEX) monitoring and analysis for aerosols and visibility, Volume III: Aerosol measurements and data bases, Final report. DRI 8743.3F Desert Research Institute, Reno, NV

Chow JC, Watson JG, Pritchett LC, Pierson WR, Frazier CA, Purcell RG (1993a) The DRI Thermal/Optical Reflectance carbon analysis system: Description, evaluation and applications in U.S. air quality studies. Atmos. Environ. 27A:1185-1201

Chow JC, Watson JG, Lowenthal DH, Solomon PA, Magliano KL, Ziman SD, Richards LW (1993b) PM_10_ and PM_2.5_ compositions in California's San Joaquin Valley. Aerosol Sci. Technol. 18:105-128

Chow JC, Watson JG, Fujita EM, Lu Z, Lawson DR, Ashbaugh LL (1994a) Temporal and spatial variations of PM_2.5_ and PM_10_ aerosol in the Southern California Air Quality Study. Atmos. Environ. 28:2061-2080

Chow JC, Fujita EM, Watson JG, Lu Z, Lawson DR, Ashbaugh LL (1994b) Evaluation of filter-based aerosol measurements during the 1987 Southern California Air Quality Study. Environ. Mon. Assess 30:49-80

Chow JC, Watson JG, Solomon PA, Thuillier RH, Magliano KL, Ziman SD, Blumenthal DL, Richards LW (1994c) Planning for SJVAQS/AUSPEX particulate matter and visibility sampling and analysis. In: Solomon PA (ed) Planning and Managing Regional Air Quality, Modeling and Measurement Studies. CRC Press, Inc., Boca Raton, FL, pp 171-216.

Chow JC, Watson JG, Lu Z, Lowenthal DH, Frazier CA, Solomon PA, Thuillier RH, Magliano KL (1996) Descriptive analysis of PM_2.5_ and PM_10_ at regionally representative locations during SJVAQS/AUSPEX. Atmos. Environ. 30:2079-2112

Chow JC, Watson JG, Lowenthal DH, Egami RT, Solomon PA, Thuillier RH, Magliano KL, Ranzieri AJ (1998) Spatial and temporal variations of particulate precursor gases and photochemical reaction products during SJVAQS/AUSPEX ozone episodes. Atmos. Environ. 32:2835-2844

Chow JC, Watson JG, Barber PW, Moosmüller H, Arnott WP, Chen L-WA (2003) Understanding thermal and optical carbon analysis methods. Desert Research Institute, Reno, NV

Chow JC, Watson JG, Chen L-WA, Arnott WP, Moosmüller H, Fung KK (2004) Equivalence of elemental carbon by Thermal/Optical Reflectance and Transmittance with different temperature protocols. Environ. Sci. Technol. 38:4414-4422

Chow JC, Watson JG, Chen L-WA, Chang M-CO, Robinson NF, Trimble DL, Kohl SD (2007) The IMPROVE_A temperature protocol for thermal/optical carbon analysis: Maintaining consistency with a long-term database. J. Air Waste Manage. Assoc. 57:1014-1023

DeBell LJ, Gebhart KA, Hand JL, Malm WC, Pitchford ML, Schichtel BA, White WH (2006) Spatial and seasonal patterns and temporal variability of haze and its constituents in the United States: Report IV. National Parks Service, Fort Collins, CO

Fitz DR, Zwicker JO (1988) Design and testing of the SCAQS sampler for the SCAQS Study, 1987. A6-077-32 AeroVironment Inc., Monrovia, CA

Fung KK (1990) Particulate carbon speciation by MnO_2_ oxidation. Aerosol Sci. Technol. 12:122-127

Fung KK, Wright B (1990) Measurement of formaldehyde and acetaldehyde using 2,4-dinitrophenylhydrazine- impregnated cartridges during the Carbonaceous Species Methods Comparison Study. Aerosol Sci. Technol. 12:44-48

Gray HA, Cass GR, Huntzicker JJ, Heyerdahl EK, Rau JA (1986) Characteristics of atmospheric organic and elemental carbon particle concentrations in Los Angeles. Environ. Sci. Technol. 20:580-589

Hand JL, Copeland SA, McDade CE, Day DE, Moore JrCT, Dillner AM, Pitchford ML, Indresand H, Schichtel BA, Malm WC, Watson JG (2011) Spatial and seasonal patterns and temporal variability of haze and its constituents in the United States, IMPROVE Report V. Cooperative Institute for Research in the Atmosphere, Fort Collins, CO

Huntzicker JJ, Johnson RL, Shah JJ, Cary RA (1982) Analysis of organic and elemental carbon in ambient aerosols by a thermal-optical method. In: Wolff GT, Klimisch RL (eds) Particulate Carbon: Atmospheric Life Cycle. Plenum Press, New York, NY, pp 79-88.

Johnson RL (1981) Development and evaluation of a thermal/optical method for the analysis of carbonaceous aerosol. Thesis, Oregon Graduate Center

Lowenthal DH, Kumar NK (2003) PM_2.5_ mass and light extinction reconstruction in IMPROVE. J. Air Waste Manage. Assoc. 53:1109-1120

Macias ES, Zwicker JO, Ouimette JR, Hering SV, Friedlander SK, Cahill TA, Kuhlmey GA, Richards LW (1981) Regional haze case studies in the southwestern United States - I. Aerosol chemical composition. Atmos. Environ. 15:1971-1986

Maenhaut W, Schwarz J, Cafmeyer J, Chi XG (2002) Aerosol chemical mass closure during the EUROTRAC-2 AEROSOL Intercomparison 2000. Nuclear Instruments & Methods in Physics Research Section B-Beam Interactions with Materials and Atoms 189:233-237

Malm WC, Sisler JF, Huffman D, Eldred RA, Cahill TA (1994) Spatial and seasonal trends in particle concentration and optical extinction in the United States. J. Geophys. Res. 99:1347-1370

Malm WC, Pitchford ML, Scruggs M, Sisler JF, Ames RG, Copeland S, Gebhart KA, Day DE (2000) Spatial and seasonal patterns and temporal variability of haze and its constituents in the United States: IMPROVE Report III. ISSN: 0737-5352-47 Cooperative Institute for Research in the Atmosphere, Colorado State University, Ft. Collins, CO

Mueller PK, Fung KK, Heisler SL, Grosjean D, Hidy GM (1982) Atmospheric particulate carbon observations in urban and rural areas of the United States. In: Wolff GT, Klimisch RL (eds) Particulate Carbon: Atmospheric Life Cycle. Plenum Publishing Corporation, New York, NY, pp 343-370.

Simon H, Bhave PV, Swall JL, Frank NH, Malm WC (2011) Determining the spatial and seasonal variability in OM/OC ratios across the US using multiple regression. Atmos. Chem. Phys. 11:2933-2949

Solomon PA, Fall T, Salmon LG, Cass GR, Gray HA, Davidson A (1989) Chemical characteristics of PM_10_ aerosols collected in the Los Angeles area. J. Air Poll. Control Assoc. 39:154-163

Table S-1. Summary of past PM_2.5_/PM_10_ studies with reconstructed mass.

| **Study or Network (Reference)/Objectives** | **Sampling Duration/Frequency/Instrument** | **Locations** | **Measurements** | **Reconstructed Mass (RM) Method ( Table 1)** |
| --- | --- | --- | --- | --- |
| Characterization of Visibility-reducing Aerosols in the Southwest: Project VISTTA  (Macias et al. 1981)  Objectives:  Determine chemical species that cause visibility impairment in the desert Southwest and the emission source types and source areas which cause visibility impairment. | Sampling 24 hours (hr)/day from 6/28/79 to 7/13/79 and 12/3/79 to 12/15/79.  A Beckman automatic dichotomous sampler (ADS) was used for PM_2.5_ and PM_15-2.5_ (coarse PM) at a flow rate of 17 L/min using Teflon-membrane filters. A separate PM_2.5_ unit equipped with an Air Industrial Hygiene Laboratory (AIHL) cyclone was followed by two filter packs: one micro-tissue quartz-fiber and the other Nuclepore-membrane filter at a flow rate of 20 L/min each. | Two sites near Page, AZ:  -Zilnez Mesa, AZ  -Copper Mine, AZ | PM_2.5_ and PM_15-2.5_ mass by gravimetry and β-gauge  Elements from Al to Pb by X-ray fluorescence(XRF) and proton induced X-ray emission (PIXE)  Anions (SO_4_^=^ and NO_3_^-^) by ion chromatography (IC)  Cation (NH_4_^+^) by spectrophotometry  Total carbon by γ-ray analysis of light elements, and elemental carbon (EC) by reflectance. | Eq. 1  RM = (NH_4_)_2_SO_4_ + NH_4_NO_3_ + 1.5OC + EC + 1.89Al + 2.14Si + 1.4Ca + 1.2K + 1.43Fe +1.25Cu + 1.24Zn + 1.08Pb  RM explained 75-93% of PM_2.5_ and 50-69% of PM_15-2.5_. |
| PM_10_ in the Los Angeles, CA area (Solomon et al. 1989)  Objectives:  Characterize PM_10_ in the South Coast Air Basin (SoCAB); document methods for future air quality modeling; and develop control measures. | Sampling 24 hr/day every sixth day during calendar year 1986.  A modified CalTech sampler with Model SA-246b Sierra Andersen PM_10_ inlet, followed by three parallel channels at a flow rate of 5.6 L/min each; using two 47 mm polytetrafluorethylene (PTFE) Teflon-membrane filters and one quartz-fiber filter. | Nine sites in SoCAB:  -Burbank  -Downtown Los Angeles  -Hawthorne  -Long Beach  -Anaheim  -Upland  -Rubidoux  -St. Nicolas Island  -Tanbark Flats (Angeles -National Forest) | PM_10_ mass by gravimetry  34 elements by XRF  Anions (Cl^-^, SO_4_^=^ and NO_3_^-^) by IC  Cations:  NH_4_^+^ by automated colorimetry (AC)  Na^+^ and Mg^++^ by flame atomic absorption spectrometry (AAS)  Carbon (OC and EC) by thermal/optical reflectance (TOR; Gray et al. 1986; Huntzicker et al. 1982; Johnson 1981) | Eq. 2  RM = SO_4_^=^ + NO_3_^-^ + NH_4_^+^ + 1.4OC + EC + 1.89Al + 2.14Si + 1.4Ca + 1.43Fe + Na^+^ + Mg^++^  RM explained 77–95% of PM_10_ for peak 24-hr mass and 86–94% for annual average mass. |

Table S-1. continued.

| **Study or Network (Reference)/Objectives** | **Sampling Duration/Frequency/Instrument** | **Locations** | **Measurements** | **Reconstructed Mass (RM) Method (Table 1)** |
| --- | --- | --- | --- | --- |
| Southern California Air Quality Study (1994a; SCAQS; Chow et al. 1994b)  Objectives:  Examine the chemical composition of PM_2.5_ and PM_10_; and develop a database for air quality modeling and control strategy development. | Sampling 4 to 7 hr on 11 episode days during summer (06/19/87–09/30/87) and 4 to 6 hr on 6 days during fall (11/11/87–12/11/87).  SCAQS sampling system (Fitz and Zwicker 1988) that included 12 channels at flow rates of 4–11 L/min for gases. Gaseous HNO_3_, and NH_3_ sampling that used denuder difference method and SO_2_ that used filter pack method.  PM_2.5_ and PM_10_ sampling at 35 L/min on Teflon-membrane, quartz-fiber, and Teflon/quartz-fiber filter packs and at 5 L/min for PM_10_ on polycarbonate-membrane filters. | Six sites during summer and fall:  -Burbank  -Downtown Los Angeles  -Hawthorne  -Long Beach  -Anaheim  -Rubidoux  Three additional sites during summer:  -St. Nicolas Island  -Azusa  -Claremont | PM_2.5_ and PM_10_ mass by gravimetry  40 elements (Na to U) by XRF  Gaseous HNO_3_ and SO_2_ by IC, and NH_3_ by AC  Anions (Cl^-^, SO_4_^=^, and NO_3_^-^) by IC  Cations:  -NH_4_^+^ by AC  -Na^+^ (PM_10_ only) by AAS  Carbon (OC and EC) by thermal magnesium oxidation (TMO; Fung 1990; Mueller et al. 1982) | Eq. 3  RM=SO_4_^=^ + NO_3_^-^ + NH_4_^+^ + 1.4OC + EC + 1.89Al + 2.14Si + 1.4Ca + 1.43Fe + trace elements  RM explained 70–80% of PM_2.5_ and 80–85% of PM_10_ mass during summer, and ~5% more during fall. Inhomogeneities of the sample deposit resulted in underestimation of geological minerals and trace metal concentrations. |
| Interagency Monitoring of PROtected Visual Environments (IMPROVE; Malm et al. 1994)  Objectives:  Establish background visibility levels and attribute light scattering and extinction to aerosols and their chemical components. | Sampling 24 hr/day from midnight to midnight every third day from March, 1988 to February, 1991 using the four-module IMPROVE sampler. | 36 IMPROVE sites in U.S. National Parks and Wilderness Areas. | PM_2.5_ and PM_10_ mass by gravimetry  25 elements by PIXE  Anions (Cl^-^, SO_4_^=^, and NO_3_^-^) by IC  Carbon (OC and EC) by IMPROVE_TOR (Chow et al. 1993a) | Eq. 4  RM =4.125S + 1.4OC + EC + 2.2Al + 2.49Si + 1.63Ca + 1.94Ti + 2.42Fe  RM explained 75-80% of PM_2.5_ mass, on average. |

Table S-1. continued.

| **Study or Network (Reference)/Objectives** | **Sampling Duration/Frequency/Instrument** | **Locations** | **Measurements** | **Reconstructed Mass (RM) Method (Table 1)** |
| --- | --- | --- | --- | --- |
| San Joaquin Valley Air Quality Study/ Atmospheric Utilities Signatures, Predictions and Experiments (SJVAQS/AUSPEX) summer study (Chow et al. 1990; 1992; 1993b; 1994c; 1996; 1998)  Objectives:  Determine temporal/spatial distributions of PM_2.5_, PM_10_, and light extinction; estimate contributions from primary and secondary sources; explain mechanisms for secondary aerosol formation and relationship between O_3­_ chemistry and secondary aerosol in central California; and enhance modeling and estimation of excess O_3_ levels in central California. | Sampling four times/day (5 to 7 hr) for five O_3_ episodes on 14 forecasted days from 07/13–08/24/90.  Desert Research Institute Sequential Gas Sampler (SGS) was used for gas sampling and Sequential Filter Samplers (SFS) were used for PM_2.5_ and PM_10_ sampling at a flow rate of 20 L/min. | One site in the San Joaquin Valley:  -Caliente  Plus nine exposure sites:  -Point Reyes  -Altamont Pass  -Pacheco Pass  -Crow’s Landing  -Academy  -Buttonwillow  -Edison  -Yosemite National Park  -Sequoia National Park  -For a total of 10 sites. | PM_2.5_ and PM_10_ mass by gravimetry  b_abs_ (light absorption) by densitometer  Gases (HNO_3_, NH_3_, and SO_2_) by AC  40 Elements (Na to U) by XRF  Anions (Cl^-^, SO_4_^=^, and NO_3_^-^) by IC  Cations:  -NH_4_^+^ by AC  -Na^+^ and K^+^ byAAS  Carbon (OC and EC) by IMPROVE_TOR (Chow et al. 1993a; 2003) | Eq. 5  RM=SO_4_^=^ + NO_3_^-^ + NH_4_^+^ + 1.4OC + EC + 1.89Al + 2.14Si + 1.4Ca 1.43Fe + Na^+^ + Cl^-^ + trace elements  RM explained more than 90% of PM_2.5_ and PM_10_ mass.  The percentage of unexplained PM_10_ mass decreased as the proportion of geological minerals increased. |

Table S-1. continued.

| **Study or Network (Reference)/Objectives** | **Sampling Duration/Frequency/Instrument** | **Locations** | **Measurements** | **Reconstructed Mass (RM) Method (Table 1)** |
| --- | --- | --- | --- | --- |
| 1995 Southeastern Aerosol Visibility Study (SEAVS; Andrews et al. 2000)  Objectives:  Test for mass closure among gravimetric, chemical, and optical measurements using four different types of samplers.  -Hypotheses for bias in mass reconstruction were:  -Errors in sampling and analysis of OC;  -Bias in the OM/OC ratio;  -Water absorption of hygroscopic inorganic species;  -Water absorption of organics; and  -Bias in the geological minerals equations | Sampling 12 hr/day (0700 to 1900 Eastern Daylight Time [EDT]) for 5 days from 7/15/95–08/25/95.  Two two-stage Stanford samplers, one Harvard-EPA annular denuder system, three micro-orifice uniform deposit impactors (MOUDIs), and one IMPROVE sampler were used for PM_2.1_ sampling except for MOUDI (PM­_1.8_­). | Look Rock Ridge, Great Smoky Mountain National Park, Tennessee | PM mass by gravimetry  38 elements (Na to U) by instrumental neutron activation analysis (INAA) for the Stanford sampler and MOUDI. 25 elements (Na to Pb) by XRF and PIXE with IMPROVE sampler  Anions (SO_4_^=^ and NO_3_^-^) by IC  Cations (NH_4_^+^) by AC  Carbon (OC and EC) by TOR (Chow et al. 1993a) for the IMPROVE sampler and by thermal manganese oxidation (Fung and Wright 1990; Mueller et al. 1982) for MOUDI and Stanford samplers | Eq. 6  RM=SO_4_^=^ + NO_3_^-^ + NH_4_^+^ + 1.4OC + EC + 1.89Al + 2.14Si + 1.4Ca + 1.2K + 1.67Ti + 1.43Fe + trace elements.  RM explained 58–68% of PM_2.1_ mass with geological minerals based on oxides, and 59-71% of PM_2.1_ mass with geological minerals estimated using principal component analysis (PCA). For 12-hour individual sample, the unexplained mass ranged -290% to 70%, attributed to measurement errors.  Unexplained mass was higher on days strongly influenced by anthropogenic emissions or nearby forest fires.  When accounting for water content (varied from 0–47%), there was still 15–23% unexplained fine PM mass.  OM/OC = 1.4 was too low for non-urban sites; using OM/OC=2.1 increased the explained mass from 70% to 77%.  Other uncertainties included changing the OC multiplier for hygroscopic organics. Subtracting OC from backup quartz-fiber filters from front filter OC overcorrects for VOC absorption. |

Table S-1. continued.

| **Study or Network (Reference)/Objectives** | **Sampling Duration/Frequency/Instrument** | **Locations** | **Measurements** | **Reconstructed Mass (RM) Method (Table 1)** |
| --- | --- | --- | --- | --- |
| Interagency Monitoring of PROtected Visual Environments (IMPROVE; Lowenthal and Kumar (2003)  Objectives:  Evaluate the accuracy, consistency, and potential biases in IMPROVE mass and light extinction reconstruction. | Sampling 24 hr/day from midnight to midnight, every third day, from 1988–1999 using the IMPROVE sampler. | 59 IMPROVE sites in U.S. National Parks and Wilderness Areas | PM_2.5_ mass by gravimetry  Elements by XRF and PIXE  Anions (Cl^-^, SO_4_^=^, and NO_3_^-^) by IC  Carbon (OC and EC) by IMPROVE_TOR (Chow et al. 1993a) | Eq. 7 (Original IMPROVE equation from Malm et al. 2000)  RM = 4.125S + 1.29NO_3_^-^ + 1.4OC + EC + 2.2Al + 2.49Si + 1.63Ca + 1.94Ti + 2.42Fe  RM explained from 61% of PM_2.5_ mass at Redwood National Park and 62% at Point Ray North Seaside to 98% at San Gorgonio Wilderness with an average of 88%. |
| EUROTRAC-2 AEROSOL Intercomparison 2000 study (Maenhaut et al. 2002)  Objectives:  Compare different aerosol instruments; evaluate the extent gravimetric PM mass could be reconstructed; as well as identify and apportion major sources of PM. | Sampling 24 hr/day starting from 0900 LST during 4/4–9/2000 and 12 hr/day from 0900–2100 LST (days) and 2100–0900 LST (nights) during 4/9–14/2000.  There were four samplers. PM_2.5_ and PM_10_ were each acquired with Whatman Q-MA quartz-fiber filters. Two Gent PM_10_ stacked filter units (SFU); one used fine (PM_2_) and coarse (PM_10-2_) with Nuclepore polycarbonate filters with 8 and 0.4 µm pore sizes, respectively, and the other Gent used a Gelman Teflo filter with 2 µm pore size for PM_10_. All samplers were operated at a flow rate of 17 L/min | Melpitz, Germany | PM mass by gravimetry  42 elements by PIXE and INAA  Anions (Cl^-^, SO_4_^=^, and NO_3_^-^) by IC  Cations (Na^+^, Mg^++^, K^+^, and Ca^++^) by IC  Carbon (OC and EC) by thermal/optical transmittance (TOT; Birch and Cary 1996) | Eq. 8  RM=SO_4_^=^ + NO_3_^-^ + NH_4_^+^+ 1.4OC + EC + 2.2Al + 2.49Si + 1.63Ca + 1.94Ti + 2.42Fe + Cl + 1.4486Na + trace elements + (K-0.6Fe) (“non-crustal” K)  RM explained 86 ± 4% and 116 ± 19% of PM_2_ and PM_10-2_ mass, respectively. |
| Interagency Monitoring of PROtected Visual Environments (IMPROVE; DeBell et al. 2006)  Objectives:  Determine the spatial and temporal distributions of PM_2.5_ and PM_10_ in U.S. Class I and selected urban areas; and attribute light scattering and extinction to aerosols and their chemical components. | Sampling 24 hr/day from midnight to midnight every third day from 2000 through 2004.  Four-module IMPROVE samplers were used at the IMPROVE sites; various multi-channel chemical speciation samplers were used at Speciation Trends Network (STN) sites. | 159 IMPROVE sites in U.S. National Parks and Wilderness areas and 84 sites in the U.S. EPA’s urban STN. | PM_2.5_ and PM_10_ mass by gravimetry  Elements by XRF (and PIXE prior to 2001)  Hydrogen (H) by proton elastic scattering analysis (PESA; only for IMPROVE sites)  Anions (Cl^-^, SO_4_^=^, and NO_3_^-^) by IC  Carbon (OC and EC) by IMPROVE_TOR protocol (Chow et al. 1993a; 2004) | Eq. 9  RM=4.125S + 1.29NO_3_^-^ + 1.8OC + EC + 2.2Al + 2.49Si + 1.63Ca + 1.94Ti + 2.42Fe  Deviations of SO_4_^=^/S from 3 suggested a systematic bias.  NO_3_^-^ losses occurred from the denuded Teflon-membrane filters, with average losses of 18–52%. |

Table S-1. continued.

| **Study or Network (Reference)/Objectives** | **Sampling Duration/Frequency/Instrument** | **Locations** | **Measurements** | **Reconstructed Mass (RM) Method (Table 1)** |
| --- | --- | --- | --- | --- |
| Interagency Monitoring of PROtected Visual Environments (Hand et al. 2011)  Revised IMPROVE Eq.  Objectives:  Evaluate the accuracy, consistency, and potential biases in IMPROVE mass and light extinction reconstruction; and evaluate the spatial and seasonal trends in aerosol mass concentration and extinction coefficients. | Sampling 24 hr/day from midnight to midnight, every third day. Data from 1988–2008 (IMPROVE) and 2000–2008 (CSN) were examined for mass reconstruction.  IMPROVE uses the four-module IMPROVE samplers; CSN uses CSN samplers and added URG 3000N samplers for carbon after 2007. | 168 IMPROVE sites and 176 CSN sites in the U.S. | PM_2.5_ and PM_10_ mass by gravimetry  25 elements (Na–Pb) by XRF (and PIXE prior to 2001)  Anions (Cl^-^, SO_4_^=^, NO_2_^-^ and NO_3_^-^) by IC  Carbon (OC and EC) by IMPROVE_TOR (Chow et al. 1993a) for measurements up to 2004 and IMPROVE_A TOR for measurements from 2005 onward (Chow et al. 2007); CSN used NIOSH_TOT (Birch and Cary 1996) prior to 2007 and IMPROVE_A_TOR protocol from 2007 onward. | Eq. 10  RM=1.375 SO_4_^=^ + 1.29NO_3_^-^ + 1.8OC + EC + 2.2Al + 2.49Si + 1.63Ca + 1.94Ti + 2.42Fe + 1.8Cl^-^  RM at IMPROVE sites typically overestimates PM_2.5_ but the difference is low (-1.14–0.6 µg/m^3^).  RM at most urban CSN sites underestimates PM_2.5_ (-0.7–5.2 µg/m^3^).  Higher PM_2.5_ concentrations and lower filter face velocity at CSN sites led to smaller negative sampling artifacts for NO_3_^-^. |
| Interagency Monitoring of PROtected Visual Environments (IMPROVE; Simon et al. 2011)  Objectives:  Establish seasonally and spatially varying OM/OC ratios in the U.S.; and address advantages and disadvantages of using multiple regression techniques to address measurement artifacts. | Sampling 24 hr/day from midnight to midnight every third day from 2002–2008.  Four-module IMPROVE samplers were used. | 186 sites in U.S. National Parks and Wilderness Areas, excluding sites with < 105 days of complete data sets per quarter; this resulted in 153 sites for regression analysis. | PM_2.5_ and PM_10_ mass by gravimetry  25 elements by XRF( and PIXE prior to 2001)  Anions (Cl^-^, SO_4_^=^, and NO_3_^-^) by IC  Carbon (OC and EC) by IMPROVE_TOR (Chow et al. 1993a) for measurements up to 2004 and the IMPROVE_A TOR protocol (Chow et al. 2007) for measurements from 2005 onward. | Eq. 11  RM = (NH_4_)_2_SO_4_ + NH_4_NO_3_ + 1.8OC + EC + 3.48Si + 1.63Ca + 2.42Fe + 1.94Ti + 1.8Cl^-^ + 1.2×(K-0.6Fe) (non-crustal K)  Overall, 90% of quarter-specific regressions yield physically reasonable coefficients. At the 50^th^ percentile, multiple regression estimated OM/OC ratios were between 1.39 and 1.83. |
